# Akwa Ibom AIDS indicator survey: Key findings and lessons learnt

**DOI:** 10.1371/journal.pone.0234079

**Published:** 2020-06-17

**Authors:** Oluwasanmi Adedokun, Titilope Badru, Hadiza Khamofu, Olubunmi Ruth Negedu-Momoh, Emem Iwara, Chinedu Agbakwuru, Akinyemi Atobatele, Mike Merrigan, Dominic Ukpong, Charles Nzelu, Gregory Ashefor, Satish Raj Pandey, Kwasi Torpey

**Affiliations:** 1 FHI 360, Abuja, Nigeria; 2 Public Health England/Nigeria, Abuja, Nigeria; 3 University of Maryland, Maryland Global Initiatives Corporation (UMB, MGIC), Abuja, Nigeria; 4 United States Agency for International Development, Abuja, Nigeria; 5 FHI 360, Durham, North Carolina, United States of America; 6 Akwa Ibom State Government, Akwa Ibom State, Nigeria; 7 Federal Ministry of Health, Abuja, Nigeria; 8 National Agency for Control of AIDS, Abuja, Nigeria; 9 University of Ghana College of Health Sciences, Accra, Ghana; University of Namibia, NIGERIA

## Abstract

**Background:**

The burden of HIV/AIDS epidemic is huge, but this varies widely by population in Nigeria. Data that could be used to guide the scale up of HIV prevention and control strategies has significant gaps. The study sought to estimate the prevalence of HIV and its associated determinants in Akwa Ibom state.

**Methods:**

Akwa Ibom AIDS Indicator Survey (AKAIS) is a population based cross-sectional survey, with a two-stage probability sampling. The survey had both behavioural and biological components. Tablet-based questionnaire was used to collect data on participant’s household information, demographics, socio-economic, and behavioral risk factors associated with HIV; while the biological component involved collection of venous blood samples for participants who were over 19months. For children aged 18months on less, capillary blood from finger prick sample was used. Participants were tested for HIV. Other biomarker tests for HIV positive participants included CD4, HIV-1 RNA viral load and incidence assays.

**Results:**

In all 15,609 people (8,963 adults aged 15 years and older (55% females), 6,646 individuals less than 15 years (51% males), from 4,313 households, participated in AKAIS. Overall, 2.8% (423 persons; 422 HIV-1 and 1 HIV-2) were found to be HIV positive. HIV prevalence was 4.8% in adults (15 years and above) and 0.4% in pediatric (< = 14 years) participants. HIV prevalence was significantly higher in females (5.6%) than males (3.7%) aged 15 years and older (p <0.001). Overall HIV incidence was 0.41%

**Conclusions:**

HIV prevalence among adults was 4.8% with an overall incidence of 0.41%. These estimates are essential to inform strategic control and prevention of HIV epidemic in Akwa Ibom state targeting the affected populations.

## Introduction

Nigeria has a generalized HIV epidemic with a HIV prevalence of 1.5% among adults 15–49 years old as at 2018. About 1,900,000 people were estimated to be living with HIV in Nigeria as at 2018. An estimated 100,000 children and adults are newly infected with HIV with about 13,000 AIDS related deaths [1]. HIV prevalence varies across states and geopolitical zones. Preliminary findings from the 2018 National AIDS Indicator and Impact Survey (NAIIS) shows the South South geopolitical zone with the highest geopolitical prevalence of 3.1%. Akwa Ibom is the South South zone with the highest prevalence of 5.5%. [2]. The United State President’s Emergency Plan for AIDS Relief (PEPFAR) has therefore prioritized Akwa Ibom state as one of the states for intensified HIV prevention and care surge activities in order to achieve epidemic control by September 2020. Epidemic control is defined as a state in which new HIV infections are lower than AIDS-related deaths. PEPFAR’s epidemic control goals derives from UNAIDS 90-90-90 goals. The 90-90-90 goals envisions that, by 2020, 90% of people living with HIV will know their HIV status, 90% of people who know their HIV-positive status will be sustained on antiretroviral treatment and 90% of people on treatment will be virally suppressed. [3, 4].

Akwa-Ibom state is in the southern region of Nigeria with a HIV prevalence of 5.5% as at 2018 [2]. In the 2014 Antenatal care (ANC) survey, Akwa-Ibom had the second highest prevalence rate of 10.8% in the country after Benue state [5]. The HIV prevalence in Akwa-Ibom state declined steadily from 12.5% in 1999 to 7.2% in 2003, this was followed by a steep rise to 10.9% in 2010, stabilizing at 10.8% in 2014 [5]. The population-based National AIDS and Reproductive Health (NARHS plus) in 2012 [6] also estimated HIV prevalence of 6.5% for Akwa Ibom state. The differences between the prevalence from different surveys (ANC 2014 and NARHS plus 2012) with less robust design therefore necessitated another survey to provide more precise estimates of HIV prevalence in Akwa Ibom state.

The purpose of Akwa Ibom AIDS Indicator Survey (AKAIS), was to generate population-based HIV estimates through state-level representative survey to inform the HIV program response in Akwa-Ibom State. The information from the survey will be valuable to program managers and policy makers as it will: provide evidence on the burden of HIV in Akwa-Ibom State, guide the scale-up of treatment and prevention services and provide the information needed for evaluation of current and future programs. AKAIS was adapted from the United States Centers for Disease Control and Prevention (CDC) HIV Impact Assessments (HIAs) which serves as a model survey. (S1 Appendix)

## Methods

### Study design

The study was a population based cross-sectional survey.

### Study area

Akwa Ibom State is located in the South-South region of Nigeria with a 2016 projected population of 5,482,177 million derived from the 2006 population census. Akwa Ibom State occupies a landmass of 8,412 square kilometers and is bounded in the north by Abia State, in the east by Rivers State, in the west by Cross River State, and in the south by the Atlantic Ocean. It has the longest coastline in Nigeria. Akwa Ibom State consists of 31 local government areas (LGAs) which are further divided into 329 political wards. The major ethnic groups are Ibibio, Annang, and Oron. The state is one of the largest producers of crude oil in Nigeria and is of major economic importance in the country [7].

### Sample size and sampling

The sample size was calculated to estimate HIV prevalence for adults ≥15 years and children aged 0–14 years. It was estimated that a sample of 4,313 households within 226 clusters would provide a representative sample of adults ≥15 years and children aged 0–14 years. Among a sample of 4,313 households, it was estimated that **16,936** adults ≥15 years and children aged 0–14 years will be recruited. With an assumption that 46% [8] of the households comprised of children aged 0–14 years the sample size target for children in this age group was estimated at **7,791** and adults ≥15 years was **9,145**.

A two-stage probability sampling technique was employed in selecting participants from a frame of eligible household residents of Akwa Ibom State. The primary sampling unit was EAs as defined by the National Population Commission (NPC) during the 2006 Nigeria Census [9]. At the first stage, 226 clusters (EAs) were selected with probability proportional to size and stratified by geographic location. At the second stage, a fixed number of households within the selected EAs were selected using systematic sampling. A complete listing of all households in selected EAs was conducted. All eligible members of the household were included in the survey.

### Community mobilization and sensitization

Before the commencement of AKAIS, extensive community mobilization and stakeholder engagements were undertaken to ensure participation, accessibility, ownership, acceptability and cooperation of communities. Existing social mobilization structures in the state was utilized to sensitize communities about the survey. Sensitization was done at the state, LGA, and village levels to ensure adequate coverage.

### Inclusion criteria, recruitment and informed consent

Household residents were eligible for study inclusion if they were: a) children aged 0 to 18 months with parental/guardian consent for Dried Blood Spot (DBS) collection onto DBS cards and rapid HIV testing; b) children aged 19 months to 9 years, children 10 to 14 years and 15-17years, with parental/guardian consent and who assent for a questionnaire, venous blood draw and HIV rapid testing; c) Women and men aged 18 years or older and mature minors aged 15 to 17 years of age who consent to a questionnaire (the Nigeria HIV testing guidelines states that children under the age of 18years who are married, pregnant, parents can independently consent to HIV testing and counseling) venous blood draw and HIV rapid testing [10].

### Data collection, management and analysis

#### AKAIS pilot testing

A pilot study was conducted in six EAs in Akwa Ibom State that were not in the main survey sampled clusters. The pilot study assisted the AKAIS team in identifying potential problems that could arise during the actual fieldwork. It also provided an opportunity to address them before the main study was done.

#### Training of field workers

Data collection was implemented by 68 teams of field workers. Each survey team comprised of eight people: one supervisor, three interviewers, one HTC service provider (counsellor), one laboratorian, one household tracker and one sample transport officer. Training of the AKAIS data collection personnel was conducted in two phases, a seven day training of trainers/facilitators and a 10-day training of field workers. A total of 46 personnel consisting of facilitators and cluster managers were trained at the first phase. A total of 716 field workers including field supervisors, interviewers, laboratory technicians, certified HTC service providers, blood specimen transport officers and household trackers were trained at the second phase. During the field work, the 68 teams were provided technical support and worked from three Central Operational Bases (COBs) delineated according to the senatorial districts in the state (NE (Uyo), NW (Ikot Ekpene) and South (Eket) senatorial districts).

#### Data collection procedures

Three questionnaires were used in the field during AKAIS: 1). a household questionnaire; 2). an individual adolescent questionnaire for individuals aged 10–14 years; and 3). an individual adult questionnaire for women and men aged 15 or older. The contents of these questionnaires were adapted from CDC-HQ HIV Impact Assessment (HIA) questionnaire (S1 Appendix).

The household questionnaire gathered basic information from the head of the household or a representative on usual members and visitors in the household, including their age, sex, education and relationship to the head of household. Information was also collected to assess household socioeconomic characteristics such as household assets’ ownership and nature of dwelling unit/abode, access to basic amenities.

The individual adult questionnaires collected information from eligible persons aged 15 years and older respectively on basic demographic characteristics, reproductive history, marriage, sexual activity, fertility, and family planning. In addition, the tool included questions regarding HIV and STI knowledge, attitudes and behaviours, HIV testing, HIV care and treatment uptake, and other health issues, such as tuberculosis, blood donation, and medical injections.

A separate questionnaire for adolescents aged 10 to 14 years was administered to eligible participants in this age group. Information was collected on demographic characteristics; HIV knowledge, attitudes and risk perception; circumcision status; HIV testing; alcohol and drug use; participation in prevention interventions; and HIV stigma perceptions.

Electronic data capture into android-based 10 inch portable tablets was employed using CSPro software. Data was transferred at the completion of each questionnaire to the central server, fully backed-up in real time by cloud technology. The CSPro software template was designed such that all questionnaire modules were available in each tablet as appropriate and several interviewers could attend to various household members concurrently. Barcodes with unique identification numbers were printed real time within the households by the survey team and placed on survey materials and laboratory specimens. Barcodes were generated for each consenting participant and served as the unique identifier. The household questionnaire had household listing schedule built into the CSPro software on the tablets and this was used to identify all usual household members and overnight guests. Power backup was assured by providing two power banks for each tablet and alternate sim cards were also provided to assure options for internet connectivity. As part of data quality assurance, validation rules were programmed into the CSPro software to automatically detect invalid responses. Skip patterns were incorporated to improve the flow of questionnaire administration. Further consistency checks for validity and completeness were performed when data were downloaded.

#### Laboratory procedures

The approach used for HIV testing in households was adapted from the National Guidelines for HIV Testing and Counselling (HTC) [11] and the WHO’s Handbook for Planning, Implementing, and Monitoring Home- Based HTC [12]. Following the interview sessions, the HIV testing for the survey involved: pre-test counselling; blood sample collection; rapid HIV testing; post-test counselling; referral and linkage to care. Rapid HIV-1/2 testing was performed at the household level on adults by following the national serial guidelines for HIV testing algorithm (Determine–Unigold–Stat-Pak) [FMOH. Federal Ministry of Health: National Guidelines for HIV Testing Services; National AIDS/ STIs Control Programme. 2017.]

**Fig 1:**
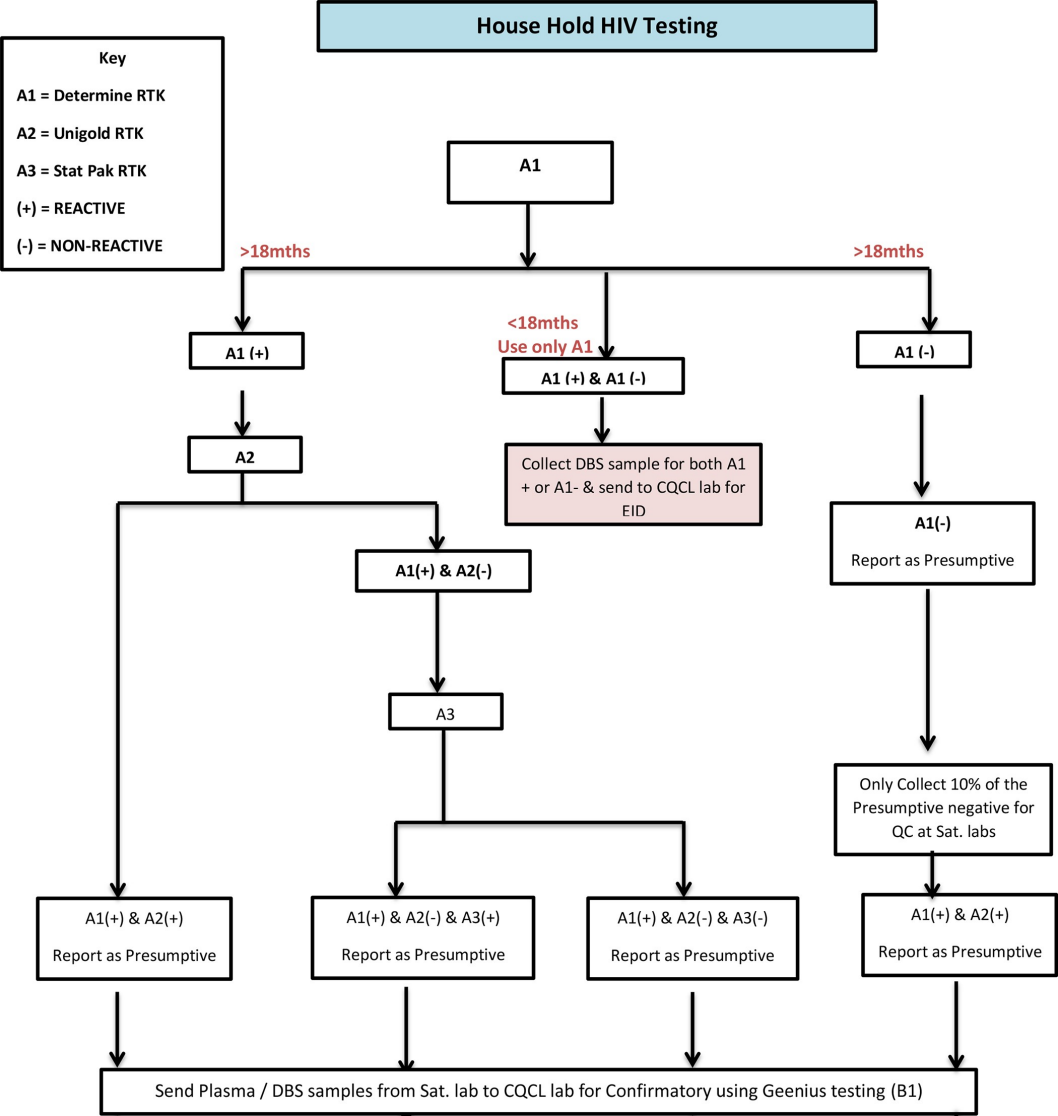
Household HIV testing algorithm, AKAIS 2017.

**Fig 2:**
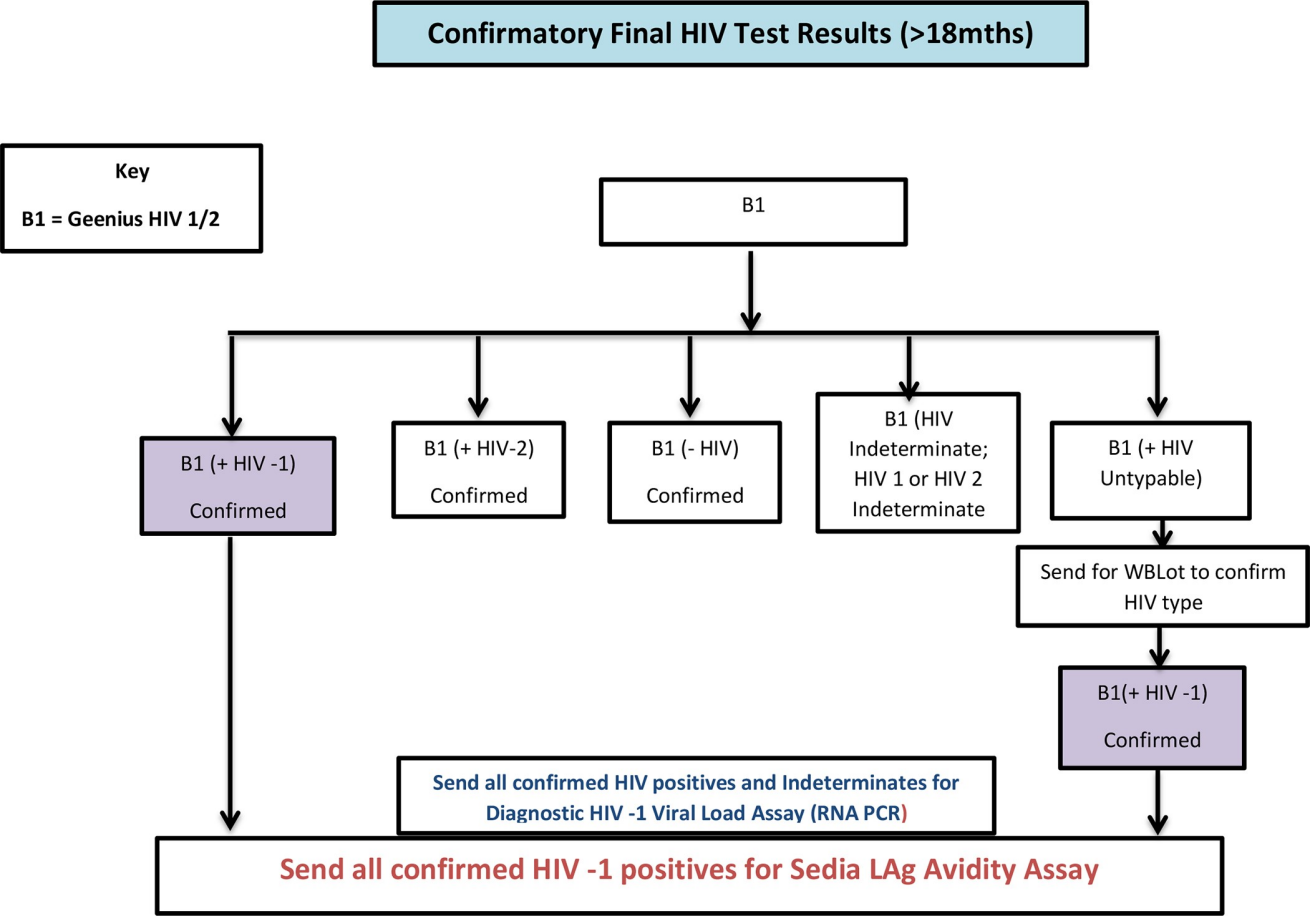
Confirmatory HIV testing algorithm (>18mnths), AKAIS 2017.

**Fig 3:**
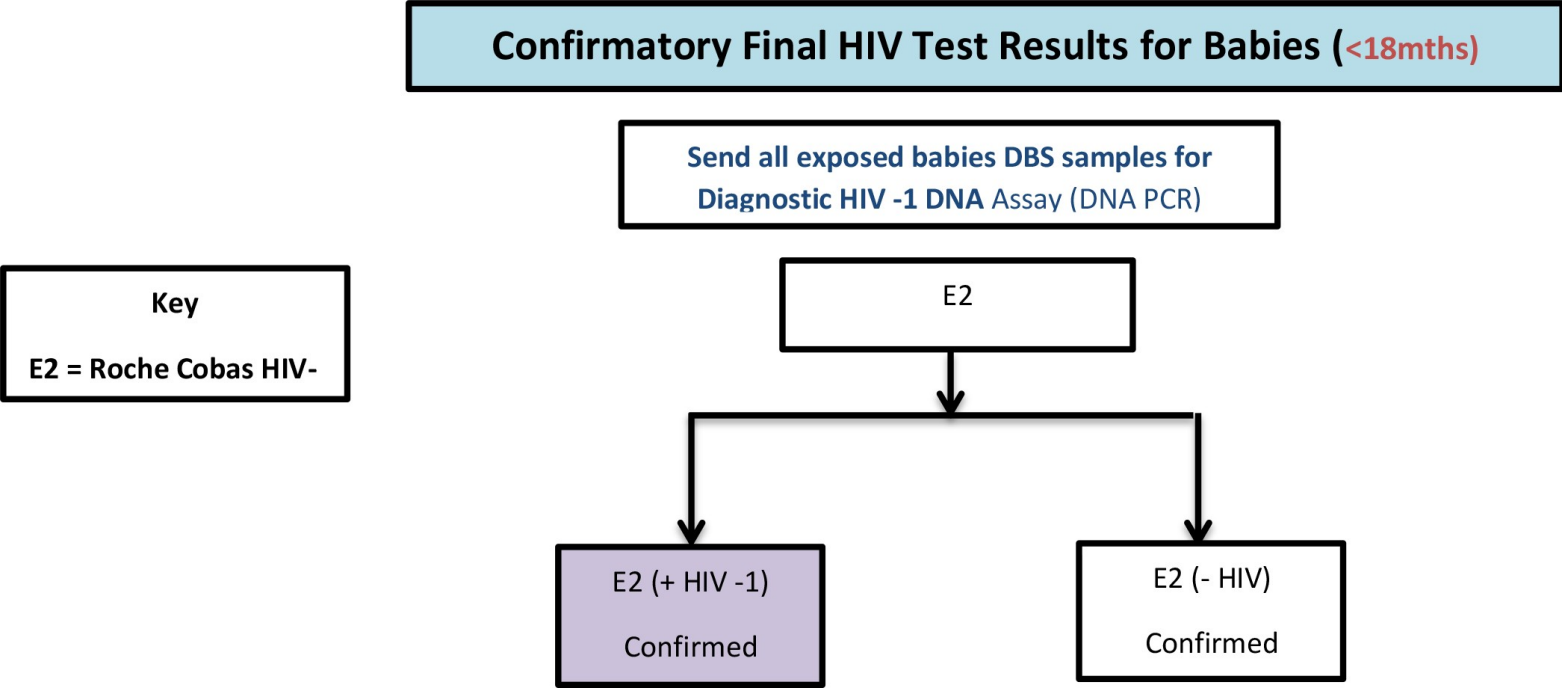
Confirmatory HIV testing algorithm for babies (<18months), AKAIS 2017.

On the field 10 ml Ethylenediaminetetraacetic (EDTA) vacutainer tubes and DBS filter paper (Munktell-TFN) were used to collect blood samples as appropriate from participants 18 months old and above who tested HIV positive for other biomarkers investigations in the laboratory, i.e. CD4, HIV -1 RNA viral load, HIV incidence assay, and quality control (QC) retesting from 10% of the same age group who tested HIV negative in the household. All venous blood samples collected were transported to a nearby selected satellite field laboratory in a triple packaging format by a Transport Officer for immediate processing for CD4 testing. The Transport Officers were persons with laboratory background and were well trained in packaging and handling of biohazard specimens/ documents for transportation to satellite laboratories. The flowchart for the laboratory test and process is shown as Figs 1,2 and 3.

The samples were then shipped to Central Quality Control Laboratory (CQCL) of University of Uyo Teaching Hospital, Uyo for HIV confirmatory test using Biorad Geenius HIV 1 / 2 kit (Biorad, France) [BIO-RAD. Geenius™ HIV 1/2 Confirmatory Assay, Bio Rad France.]. Samples that were confirmed HIV positives were further tested for HIV-1 RNA Viral Load using Cobas Roche Ampliprep / Taqman analyzer version 2.0 on the 96 CAP- CTM analyzer in line with manufacturer instructions using plasma aliquot of 1.0ml [Roche. Cobas AmpliPrep-Cobas TaqMan HIV-1 test, v.2.0, Package insert. Roche Molecular Systems, Branchburg, NJ. 2008]

Plasma eluates from samples of participants over 15 years of age were tested with the LAg-Avidity enzyme immunoassay (EIA) to determine HIV incidence using a recent infection testing algorithm (RITA) at the CQCL of University of Uyo Teaching Hospital. A total of 370 eligible plasma samples with accompanying viral load results were tested for recent HIV infection out of the 394 HIV-1 seropositive samples of persons 15 years and above. To minimize potential misclassification on the assay, the viral load cut off of <1000 copies/ml was applied to the LAg assay. The Sedia Bioscience LAg assay kit (Sedia Biosciences Corporation Portland, Oregon, USA) was used to determine HIV incidence because of its ability to be used to assay both plasma and DBS specimens. HIV-incidence calculation was performed using the Sedia LAg data management sheet and HIV incidence calculator developed by CDC [13]. HIV Incidence is defined as the number of new HIV infections occurring in a population, usually expressed as a rate of infection per person per unit of time [14].

### Data analysis

Sampling weights were calculated in order to adjust for the sample design. Weighted proportions alongside 95% confidence intervals were reported. Chi-square test (or Fisher’s exact test in cases of small subgroup sample size) was used to elicit associations between categorical variables. Multivariable logistic regression model was conducted to determine associations with HIV infection. P-value less than 0.05 were considered to be significant for all analysis. Statistical analyses were performed using Stata 12.0 (StataCorp, 2012, Stata Statistical Software: Release 12.0, College Station, TX: StataCorp LP).

### Ethical approval

The AKAIS protocol was approved by the FHI 360 Protection of Human Subjects Research Ethics Committee, North Carolina, U.S.(Approval #797214), the Akwa Ibom State Ministry of Health Ethics Committee (Approval #MH/PRS/99/VOL.VII/506), the University of Uyo Teaching Hospital Review Committee (Approval # UUTH/AD/S/96/VOL.XIV/482) and the University of Nigeria Nsukka Teaching Hospital Health Research Ethics Review Committee (Approval #UNTH/CSA/329/OL5).

Participation in the study was voluntary. Consent was informed and signed. Consent was obtained from parents or guardians of children 0-18months. For children aged 19months to 17years, parental/guardian consent in addition to assent was sought. Participants aged 18 years and above provided consent before participation.

## Results

### Respondents characteristics

**Fig 4:**
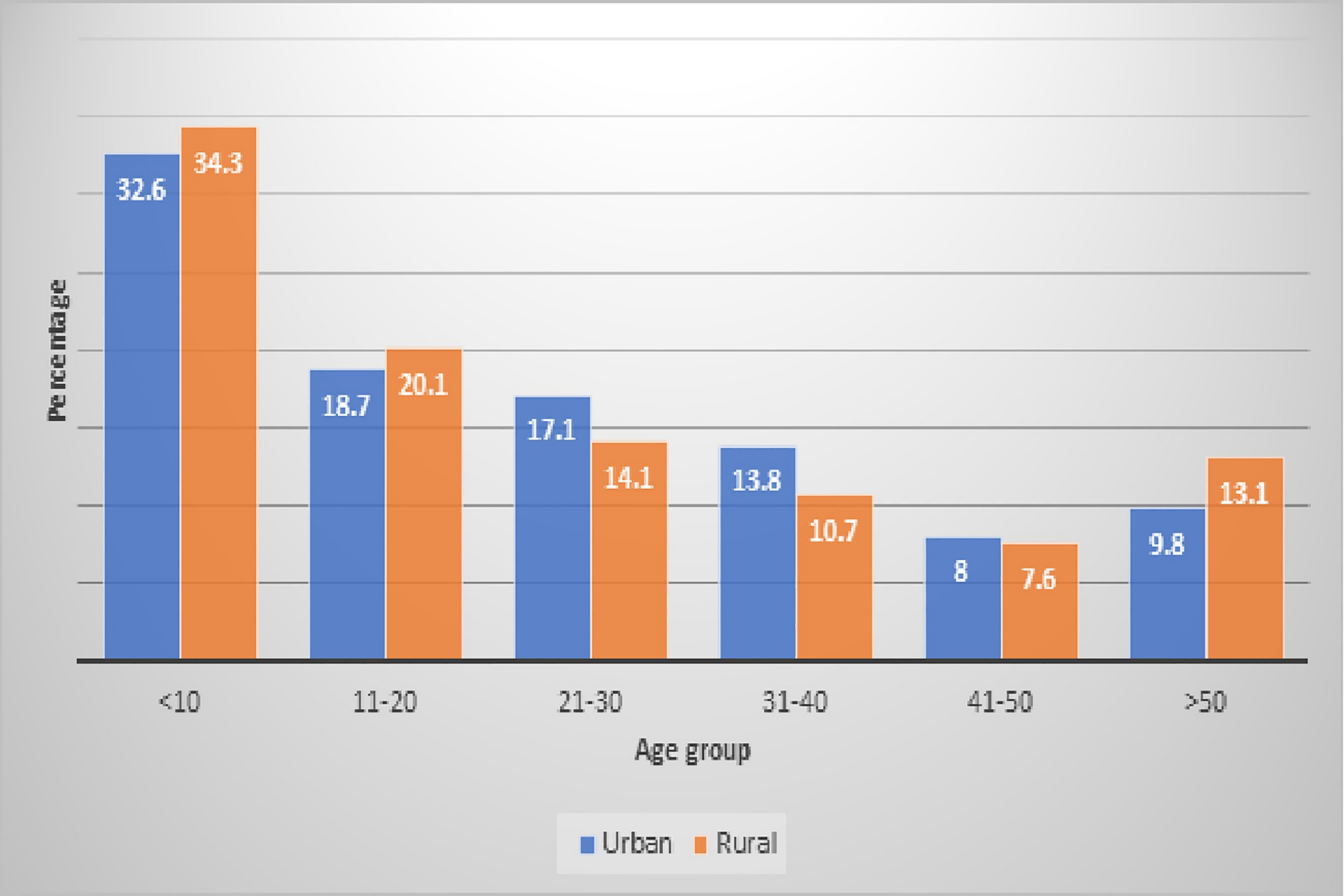
Household population by age category, AKAIS 2017.

A total of 4,313 household questionnaires were analyzed, representing the numbers of households in the survey. Table 1 shows the distribution of household demographics and composition by place of residence. Most (90.8%) of the respondents that completed the household questionnaires were the head of households. A total of 16,994 people were listed from the selected households. There were more females, 52.6%, than males, 47.4% in the overall household make-up (Table 1). Children below 10 years were more in the households than other age categories (Fig 4).

**Table 1:** Demographic and household composition (unweighted), AKAIS 2017.

Variable	Urban		Rural		Totals	
	n	%	n	%	N	%
Number of households surveyed	1,364	31.6	2,949	68.4	4,313	100
**Respondent**						
Head of household	1,214	89.0	2,703	91.7	3,917	90.8
Representative of head of household	150	11.0	246	8.3	396	9.2
**Gender of household heads**						
Male	909	67.0	1,849	61.8	2422	63.5
Female	455	33.0	1,100	38.2	1393	36.5
Average household size (mode)	4		4		4	
Total number of individuals surveyed (eligible)	5,157	30.3	11,837	69.7	16,994	100
**Gender of household members**						
Male	2,434	47.2	5,623	47.5	8,057	47.4
Female	2,723	52.8	6,214	52.5	8,937	52.6

### Survey response rate

Of 9,666 eligible individuals aged 15 years and older, 8,963 (92.7%) completed individual interviews, and 8,306 (92.7%) provided a valid blood specimen. A total of 7,325 children aged 0 month to 14 years were eligible for a blood draw. Of these, 6,593 (90.0%) provided a valid blood specimen.

### HIV prevalence

The overall prevalence of HIV in Akwa Ibom state from the survey is 2.8%. The prevalence of HIV in children aged 0–9 years and adolescents aged 10–14 years were 0.4%, and 0.6% respectively (Table 2). In children aged 0–9 years, HIV prevalence was 0.2% in the urban areas and 0.4% in the rural areas (p = 0.26). Among adolescents aged 10–14 years HIV prevalence was 0.0% in the urban area and 0.9% in the rural area (p = 0.04).

**Table 2:** HIV Prevalence disaggregated by place of residence and gender for children 0–9 years and adolescents 10–14 years, AKAIS 2017.

Variables	Children aged 0–9 years			Adolescents aged 10–14 years		
	n	% HIV positive	95% C.I	N	% HIV positive	95% C.I
Point prevalence	4,828	0.4	0.2–0.6	1,765	0.6	0.3–1.2
Gender						
Male	2,409	0.3	0.2–0.7	740	0.8	0.4–1.6
Female	2,419	0.4	0.2–0.8	825	0.4	0.2–1.2
Place of residence						
Urban	1,382	0.2	0.1–0.7	524	-	-
Rural	3,446	0.4	0.3–0.7	1,241	0.9	0.5–1.7

A total of 8,306 people aged 15 years and older were tested for HIV in Akwa-Ibom state (Table 3). Of the 8,963 adults aged 15 years and older interviewed, point prevalence of HIV was 4.8% (95% CI 4.2–5.3). HIV prevalence was about 5.1% in rural areas, compared to about 3.9% in urban areas in this age group (p = 0.04).

**Table 3:** HIV Prevalence among adults aged 15 years and older by selected socio-demographics AKAIS 2017.

Variable	Total tested	Number HIV positive	% HIV positive	95% C.I
Overall	8,306	394	4.8	4.2–5.3
**Residence**				
Urban	2,534	102	3.9	3.2–4.8
Rural	5,772	292	5.1	4.5–5.9
**Sex**				
Male	3,715	136	3.7	3.0,-4.4
Female	4,591	258	5.6	4.9–6.4
**Age group**				
15–19	1,297	20	1.5	1.0–2.3
20–24	1,075	50	4.7	3.6–6.0
25–29	1,043	57	5.3	4.1–7.0
30–34	925	65	7.3	5.7–9.4
35–39	789	61	7.6	5.8–9.7
40–49	1,184	74	6.3	5.0–7.9
≥50	1,993	67	3.3	2.6–4.3
**Educational status**				
No education	828	31	3.8	2.7–5.4
Primary	2,437	152	6.3	5.3–7.6
Secondary	3,953	167	4.2	3.6–4.8
Tertiary	1,066	27	2.5	1.7–3.6
Missing	22	17	*	
**Marital status**				
Single	2,996	93	3.1	2.5–3.8
Married	3,641	176	4.8	4.1–5.7
Cohabiting	180	6	3.4	1.5–7.5
Previously married	1,452	102	7.0	5.8–8.5
**Employment status**				
Done any work in the last 12 months for which cash or kind was received as payment	3,204	153	4.8	4.0–5.7
No work was done in the last 12 months for which cash or kind was received as payment	5,082	224	4.4	3.8–5.1

*Respondents with missing values were removed from this analysis

HIV prevalence was significantly higher in females (5.6%) than males (3.7%) aged 15 years and older (p<0.001). Prevalence was highest among previously married adults (7.0%). HIV prevalence were 4.8%, 3.4%, 3.1% among married, co-habiting, and never married adults respectively (p<0.001).

A total of 8,926 people aged 15 years and older responded to the question on ever had sex. Over 90% in this age group had ever had sex. This proportion was higher among females (91.9%) than males (89%) (p<0.001). Furthermore, of the people aged 15 years and older who had ever had sex, about 99% said they had ever had vaginal sex, while 1.8% said that they had ever had anal sex. Those who ever had vaginal or anal sex were not mutually exclusive. HIV prevalence was higher among those who had ever had sex (3.5%) compared to those who had never had sex (0.5%) (p<0.001).

A multivariable logistic regression of factors associated with HIV infection indicated that HIV infection was associated with sex, age, education [primary-, secondary], marital status [previously married vs married]; (Table 4).

**Table 4:** Factors associated with HIV infection.

	Crude OR (95% C.I)	P-value	Adjusted OR (95% C.I)	P-value
**Location**				
Urban	1		1	
Rural	1.27 (1.01–1.59)	0.042	1.13 (0.88–1.46)	0.326
**Senatorial district**				
Uyo	1		1	
Ikot Ekpene	1.10 (0.85–1.42)	0.490	0.99 (0.75–1.31)	0.972
Eket	1.49 (1.16–1.91)	0.002	1.34 (1.02–1.75)	0.032
**Sex**				
Male	1		1	
Female	1.57 (1.27–1.94)	<0.001	1.57 (1.25–1.99)	<0.001
**Age (years)**				
15–19	1		1	
20–24	3.11 (1.84–5.27)	<0.001	1.99 (1.13–3.52)	0.018
25–29	3.69 (2.20–6.18)	<0.001	2.42 (1.36–4.32)	0.003
30–34	4.83 (2.90–8.03)	<0.001	3.12 (1.74–5.62)	<0.001
35–39	5.35 (3.20–8.94)	<0.001	3.30 (1.80–6.08)	<0.001
40–49	4.26 (2.58–7.02)	<0.001	2.33 (1.27–4.28)	0.007
≥50	2.22 (1.34–3.68)	0.002	1.22 (0.65–2.31)	0.534
**Educational status**				
Tertiary	1		1	
None	1.50 (0.89–2.53)	0.132	1.62 (0.92–2.86)	0.097
Primary	2.56 (1.69–3.88)	<0.001	2.45 (1.58–3.80)	<0.001
secondary	1.70 (1.12–2.56)	0.012	1.89 (1.24–2.89)	0.003
**Marital status**				
Married	1		1	
Never married	0.64 (0.50–0.83)	0.001	1.05 (0.78–1.43)	0.738
Previously married	1.51 (1.18–1.94)	0.001	1.89 (1.44–2.50)	<0.001
Cohabiting	0.68 (0.30–1.55)	0.359	0.61 (0.27–1.42)	0.256
**Ever had sex**				
No	1		1	
Yes	0.10 (0.04–0.26)	<0.001	4.56 (1.60–12.99)	0.004
**HIV risk perception**				
No	1		1	
Yes	2.27 (1.83–2.83)	<0.001	2.16 (1.72–2.71)	<0.001
Don’t know	1.53 (1.07–2.21)	0.021	1.56 (1.08–2.26)	0.019

### HIV incidence

Annual HIV incidence among adults ages 15 years and older was 0.41% (Table 5). This translates to 13,000 new cases of HIV infections annually in persons 15 years and older in Akwa Ibom. The HIV incidence rates were similar in females and in males (0.41 percent among females and 0.42 percent among males). The HIV incidence in people aged 15–19 was 0.84 percent, translating to 5,000 estimated new infections in this age group; this accounts for nearly half of the new infections occurring in age groups 15 years and older. Incidence in this age group (15–19 years) is higher in males (1.46 percent) than in females (0.96 percent).

**Table 5:** HIV Incidence (percentage) and number of new infections by age and sex among adults 15 years and older.

Age group (years)	HIV Incidence % (95% C.I)	Estimated No. of New Infections*
**Overall**	0.41 (0.16, 0.66)	13000 (5000–21000)
**Sex**		
Male	0.42 (0.05, 0.79)	7000 (800–13000)
Female	0.41 (0.08, 0.74)	6000 (1200–12000)
**Age group**		
15–19	0.84 (0.00, 1.78)	5000 (0, 11000)
20–24	0.00 (N.D)	-
25–29	0.28 (0.00, 0.83)	1000 (0, 4000)
30–34	0.35 (0.00, 1.05)	1000 (0, 3000)
35–39	0.76 (0.00, 1.82)	2000 (0, 5000)
40–49	0.53 (0.00, 1.27)	2000 (0, 6000)
≥ 50	0.31 (0.00, 0.74)	2000 (0, 4000)

N.D: Not Determined

*numbers rounded off to the nearest thousand

## Discussion

After the first case of AIDS was reported in Nigeria, progress has been made in monitoring HIV prevalence estimates using sentinel surveillance and population-based surveys. The use of National HIV Sentinel Survey (HSS) among pregnant women attending antenatal clinics for assessing the epidemic was adopted by the Nigerian government in reporting HIV prevalence in line with WHO guidelines [5]. AKAIS presents a paradigm shift in estimating HIV prevalence in Nigeria because it focuses on general population rather than sentinel surveillance. Previous HIV prevalence estimates were largely based on ANC surveillance surveys amongst pregnant women in sentinel sites. ANC surveillance surveys assumes that prevalence among pregnant women is a good approximation of prevalence among adult population of men and women 15–49 years old. There are concerns however about the representativeness of ANC surveys for the pregnant women in the population. In Nigeria where ANC attendance rates are low, consequently undermining the accuracy of ANC prevalence estimates [15, 16, 17]. This limitation can be addressed through the use of more robust methodologies. A previous population-based survey providing HIV prevalence estimate for Akwa Ibom state was the National AIDS and Reproductive Health Surveys (NARHS plus) in 2012, with prevalence rate of 6.5% [6]. While this survey was also focused on the general population and employed serological testing for HIV prevalence estimation, AKAIS differs in that it provided population-based estimate of HIV incidence in addition to providing a more recent estimate of HIV prevalence in Akwa Ibom state. The findings of this survey corroborate with previous studies which shows that prevalence has declined in the state compared with pre-millennium rates [5]. AKAIS findings are also similar to the findings of the National AIDS Indicator and Impact Survey (NAIIS) which was conducted about a year after. Both studies were cross sectional in design, with two-stage probability sampling. A total of 8,306 adults were tested for HIV in AKAIS while NAIIS tested 4,403 adults. The preliminary data from NAIIS found an HIV prevalence of 5.5% (5% CI 4.7–6.3).There were significant overlaps in the confidence intervals for both studies, thus indicating applicability to a similar population.

AKAIS is the first population-based survey in any state in Nigeria to provide estimates of HIV incidence. Previous mathematical modelling estimates of HIV incidence in Akwa Ibom state using SPECTRUM software showed incidence rates of 0.8 to 1.4% between 1995 and 2013 [18]. The study found an HIV incidence of 0.41% amongst persons 15 years and older from the AKAIS which translates to about 13,000 new infections in 2017. This is about half of the 2013 estimates from SPECTRUM [18].

In this survey, the following factors were associated with HIV infection sex, age, education, marital status, history of sexual exposure and risk perception. Females had higher odds of HIV infection compared to males. This finding is consistent with previous surveys which shows a feminization of the epidemic in Nigeria [19]. Plausible reasons identified in the literature for the feminization of the epidemic in Nigeria include poverty, child marriage, gender-based violence, gender norms, disabilities, harmful traditional practices as well as human rights, legal and political factors [19].

AKAIS also demonstrated higher odds of HIV infection with advancing age amongst persons 15–39 years with gradually declining odds from age forty. The age groups with higher odds of HIV infection approximates fairly well with the reproductive age group in Nigeria which are sexually active [19]. The highest odds of HIV infection was found in those with primary education compared to tertiary education, secondary education and no formal education. This finding is similar to the results of NARHS plus 2012 in which HIV prevalence was highest amongst respondents with primary and secondary education and lowest amongst those with quoranic education and those with no formal education [6]. This may be due to inadvertent low risk perception amongst those with limited formal education and consequent increased risky behavior.

Compared to those who never married or currently married, previously married persons (widowed, divorced or separated) were twice as likely to be HIV infected. We posit that some of those that were previously married on the one hand may have lost their spouse to HIV. On the other hand, being free from a formal sexual relationship may predispose them to risky sexual behavior including having multiple sexual partners or casual partners [20, 21].

Not unexpectedly, those who had ever had sex were more likely to have HIV infection. Considering that 99% of those who ever had sex had ever had vaginal sex, this finding is consistent with the HIV epidemiology in Nigeria which shows that about 80% of HIV transmission is through heterosexual relationships [19].

Young person ages 15–19 years accounted for about half of new infections. Nigeria Demographic and Health Surveys 2018 documented that only 11.5% of females and 12.2% of males 15–19 years had comprehensive knowledge of HIV/AIDS. Whereas, persons in this age group practice high risk sexual behavior including multiple sexual partnerships, sexual intercourse with casual partners and low condom use [22]. Low level of comprehensive knowledge about HIV coupled with multiple sexual partners, casual sex and low condom use may largely account for the high HIV incidence in this age group. HIV prevention interventions should be more focused on these young persons.

This survey findings provides a good empirical data on the prevalence of HIV in Akwa Ibom state; and contribute to the understanding of other behavioral and sociodemographic factors in the study population. The data could also be used to guide the scale up of HIV prevention and control strategies towards achieving epidemic control in Akwa Ibom state.

The limitation of AKAIS is mainly the cross-sectional nature of the survey, which only provides a snapshot of the HIV prevalence in Akwa Ibom state.

We learnt some critical lessons in the course of implementing this survey which may inform the conduct of future surveys. Lessons learnt include: 1). Decentralization of survey support and implementation teams to operational bases closer to the surveyed households reduced response time and cost of technical support in addition to simplifying commodity logistics; 2). Use of electronic data collection with real time data synchronization to the server facilitated timely review of the data and feedback to field teams for corrective action while data collection was still ongoing; 3). Prior determination of predominant Global System for Mobile Communication (GSM) networks and provision of sim cards for alternate networks facilitated seamless data transmission; 4). Provision of at least two power banks for each Android tablet device used for field data collection significantly minimized interruptions to data collection as a result of power constraints; 5). Use of persons with laboratory background as sample transport officers ensured the integrity of the samples were maintained as these transport officers appreciated the importance of maintaining the cold chain while also providing support to the staff at the satellite labs in sample processing; 6) Persons who served as household mappers during the pre-survey household mapping and listing exercise, also doubled as household trackers during the survey, this further facilitated entry into the households as these persons had built initial rapport with the households during the household mapping and listing exercise.

In conclusion, AKAIS provides more precise estimates of HIV prevalence as well as incidence of HIV in Akwa Ibom state. The risk factors to infection and the disease burden in the state are now better understood and should be used for improved targeting, programming and budgeting for HIV control in Akwa Ibom state. The key lessons learnt will also inform future surveys.

## Supporting information

S1 Appendix (ZIP)

S1 Dataset (ZIP)
