# Supplementary material for: Akwa Ibom AIDS indicator survey: Key findings and lessons learnt
Source: PLoS One. 2020 Jun 17;15(6):e0234079. doi: 10.1371/journal.pone.0234079 (PMC7299391; doi:10.1371/journal.pone.0234079)
Supplement: S1 Appendix — (ZIP) [file pone.0234079.s001.zip › Questionnaires/AKAIS Adult Individual Questionnaire.pdf]

AKAIS ADULT INDIVIDUAL QUESTIONNAIRE (ALL Participants ≥15 years of age)

Contents

LANGUAGE AND INFORMED CONSENT 2
MODULE 1: RESPONDENT BACKGROUND 5
MODULE 2: MARRIAGE 10
MODULE 3: REPRODUCTIVE HEALTH 16
MODULE 4: CHILDREN 34
MODULE 5: SEXUAL ACTIVITY 48
MODULE 6: HIV/AIDS KNOWLEDGE AND ATTITUDES 57
MODULE 7: HIV/AIDS TESTING 60
MODULE 8: HIV STATUS, CARE AND TREATMENT 66
MODULE 9: TUBERCULOSIS AND OTHER HEALTH ISSUES 78
MODULE 10: BLOOD SAFETY AND MEDICAL INJECTIONS 81
MODULE 11: NON-PRESCRIPTION DRUG USE/ALCOHOL USE 87

LANGUAGE AND INFORMED CONSENT

NO.	VARNAME	QUESTIONS	CODING CATEGORIES	SKIPS/FILTERS	CORE/SUPPLEMENT	AKDAIS ONLY	NOTES
1		FIRST NAME OF THE PARTICIPANT FROM HOUSEHOLD ROSTER<br><br>CONFIRM NAME WITH PARTICIPANT	FIRST NAME: ________________________				
2		AGE OF PARTICIPANT FROM HOUSEHOLD ROSTER<br><br>ASK PARTICIPANT: "How old were you at your last birthday?"<br><br>*IF AGE DIFFERENT FROM HOUSEHOLD ROSTER, PLEASE VERIFY CORRECT AGE OF PARTICIPANT*	AGE: _______________________	MUST BE >14 YEARS			
3		SCAN BARCODE OF PARTICIPANT					

4		RECORD SEX OF THE RESPONDENT	MALE = 1<br>FEMALE = 2				
5		TO INTERVEIWER: FOLLOW THE NEXT STEPS<br>1) ASSESS (**NAME**)'S ELIGIBILITY (LANGUAGE, HEARING ABILITY, COGNATIVE ABILITY)<br>2) Consent (**NAME**)					
6		FOR THE INTERVIEWER, DON'T READ OUT LOUD<br><br>Is (**NAME**) eligible for the survey based on assessment (STEP 1)?	YES = 1<br>NO = 2	IF YES →8			
7		Reason for ineligibility:	HEARING DISABILITY=1<br>DOES NOT SPEAK A LANGUAGE THE SURVEY TEAM CAN ACCOMMODATE=2<br>VISUAL IMPAIRMENT=3<br>COGNITIVE DISABILITY=4<br>OTHER=98<br>(SPECIFY)	END INTERVIEW			
8		FROM CONSENT FORM:<br><br>Did you consent to take part?	YES = 1<br>NO = 2	IF YES, → 10			

9		What are the reason that you do not want to participate in the survey?<br><br>SELECT ALL THAT APPLY	NO TIME= 1<br>NOT COMFORTABLE WITH INTERVIEW= 2<br>DOESN'T LIKE QUESTIONS ON SEX= 3<br>DOESN'T LIKE BLOOD DRAW= 4<br>DOESN'T WANT TO GET RESULTS= 5<br>WORRIES ABOUT CONFIDENTIALITY= 6<br>DOESN'T WANT TO BE TESTED FOR HIV=7<br>ALREADY KNOWS HIV POSITIVE=8<br>OTHER (SPECIFY)=96<br>___________________________________<br>REFUSES TO SAY= 99	END INTERVIEW			
10		What Language do you prefer for our discussion today?	ENGLISH=1<br>ANNANG=2<br>IBIBIO=3<br>ORO=4<br>PIDGIN=5		C		

MODULE 1: RESPONDENT BACKGROUND

NO.	VARNAME	QUESTIONS	CODING CATEGORIES	SKIPS/FILTERS	CORE/SUPPLEMENT	AKAIS ONLY	NOTES
THANK YOU FOR AGREEING TO PARTICIPATE IN THIS SURVEY. THE FIRST SET OF QUESTIONS IS ABOUT YOUR LIFE IN GENERAL. AFTERWARDS, WE WILL MOVE ON TO OTHER TOPICS.							
101		What is your ethnic group/tribe?	ANNANG=1<br>IBIBIO=2<br>ORON=3<br>EFIK=4<br>YORUBA=5<br>IGBO = 6<br>HAUSA = 7<br>OTHER (SPECIFY) = 96<br>____		S		
102		What is your religion?	ISLAM= 1<br>CHRISTIAN=2<br>TRADITIONAL= 3<br>NO RELIGION=4<br>OTHER (SPECIFY)=96<br>____<br>DON'T KNOW=98<br>REFUSED TO SAY=99		S		
102B		What is your denomination?	PROTESTANT=1<br>CATHOLIC=2<br>PENTECOSTAL=3				

NO.	VARNAME	QUESTIONS	CODING CATEGORIES	SKIPS/FILTERS	CORE/SUPPLEMENT	AKAIS ONLY	NOTES
			OTHER (SPECIFY)=96<br>____<br>DON'T KNOW=98<br>REFUSED TO SAY=99				
103		Have you ever attended school?	YES = 1<br>NO = 2<br>DON'T KNOW = 98<br>REFUSED TO SAY = 99	IF NO, DK, REFUSED<br>→107	C		
104		Are you enrolled in school?	YES = 1<br>NO = 2<br>DON'T KNOW = 98<br>REFUSED = 99	IF DK, REFUSED<br>→107	C		

NO.	VARNAME	QUESTIONS	CODING CATEGORIES	SKIPS/FILTERS	CORE/SUPPLEMENT	AKAIS ONLY	NOTES
105		What is your highest level of education attained?<br>READ ALL RESPONSES ALOUD	NONE = 1<br>SOME PRIMARY = 2<br>PRIMARY = 3<br>SOME SECONDARY = 4<br>SECONDARY = 5<br>POST-SECONDARY/TERTIARY = 6<br>QUR'ANIC ONLY=7<br>DON'T KNOW=98<br>REFUSED TO SAY=99	IF DK, REFUSED →107	C		
106		What is the highest class/form you completed at that level?	CLASS/FORM _____<br>DON'T KNOW = 98<br>REFUSED = 99	SKIP IF QUR'ANIC SCHOOL	C		
107		Have you done any work in the last <u>12 months</u> for which you received cash or in kind as payment?	YES = 1<br>NO = 2<br>DON'T KNOW=98<br>REFUSED TO SAY=99	IF NO, DK REFUSED → NEXT MODULE	C		
108		Have you done any work in the last <u>seven days</u> for which you received cash or in kind as payment?	YES = 1<br>NO = 2<br>DON'T KNOW=98<br>REFUSED TO SAY=99		C		
109		What do you do for a living?	DIRECTOR/UPPER MANAGEMENT=1<br>OTHER MANAGEMENT=2<br>SALES MANAGER/REPRESENTATIVE/<br>INSURANCE BROKER=3				

NO.	VARNAME	QUESTIONS	CODING CATEGORIES	SKIPS/FILTERS	CORE/SUPPLEMENT	AKAIS ONLY	NOTES
		PROBE FOR ALL ANSWERS, TICK ALL THAT APPLY	PROFESSIONAL/SPECIALIST=4<br>SELF EMPLOYED/OWN SMALL BUSINESS=5<br>SELF EMPLOYED (INFORMAL SECTOR /HAWKERS/VENDORS ETC.)=6<br>BLUE COLLAR SKILLED & SEMI SKILLED=7<br>UNSKILLED=8<br>CLERK/CLERICAL=9<br>CIVIL SERVANT=10<br>FARMER/FORESTRY/FISHING/MINING=11<br>HOUSEWIFE=12<br>PENSIONER/RETIRED=13<br>UNEMPLOYED=14<br>STUDENT=15<br>OTHER(SPECIFY)=96<br>____<br>DON'T KNOW=98<br>REFUSED TO SAY=99				
110		How long have you been living in this community?<br><br>IF LESS THAN ONE YEAR, RECORD '00" YEARS	NUMBER. OF YEARS _ _<br><br>ALWAYS = 95<br>VISITOR = 97<br>DON'T KNOW = 98<br>REFUSED TO SAY = 99				

NO.	VARNAME	QUESTIONS	CODING CATEGORIES	SKIPS/FILTERS	CORE/SUPPLEMENT	AKAIS ONLY	NOTES
111		In the last 12 months, have you travelled to anywhere outside your community?	YES=1<br>NO=2<br>DON'T KNOW = 98<br>REFUSED TO SAY = 99	IF NO→201			
112		How many times did you travel and slept away in the last 12 months?	NUMBER OF TIMES_ _<br><br>DON'T KNOW = 98<br>REFUSED TO SAY = 99				
113		How many of these trips were you gone for one month or greater?	NUMBER OF TIMES_ _<br><br>DON'T KNOW = 98<br>REFUSED TO SAY = 99	CAN'T BE >112			

MODULE 2: MARRIAGE

NO.	VARNAME	QUESTIONS	CODING CATEGORIES	SKIPS/FILTERS	CORE/SUPPLEMENT	AKAIS ONLY	NOTES
**[INTERVIEWER READ]:** NOW I WOULD LIKE TO ASK YOU ABOUT YOUR CURRENT AND PREVIOUS RELATIONSHIPS AND/OR MARRIAGES.							
201		Have you ever been married or lived with a partner as if married?	YES = 1<br>NO = 2<br>REFUSED TO SAY = 99	IF NO, REFUSED → 301	C		
202		At what age were you first married?	AGE AT MARRIAGE: ___ ___<br>DON'T KNOW = 98<br>REFUSED TO SAY = 99		S		
203		Have you ever been widowed? That is, did a spouse ever pass away while you were still married or living with them?	YES = 1<br>NO = 2<br>REFUSED TO SAY = 99		S		
204		What is your current marital status? Are you married, living together with someone as if married, widowed, divorced, or separated?	MARRIED = 1<br>LIVING WITH A PARTNER AS IF MARRIED = 2<br>WIDOWED = 3<br>DIVORCED = 4<br>SEPARATED =5<br>REFUSED TO SAY = 99	WIDOWED, DIVORCED, SEPARATED, REFUSED → 301	C		

NO.	VARNAME	QUESTIONS	CODING CATEGORIES	SKIPS/FILTERS	CORE/SUPPLEMENT	AKAIS ONLY	NOTES
**[INTERVIEWER READ]:** NOW I WOULD LIKE TO ASK YOU SEVERAL QUESTIONS ABOUT YOUR CURRENT SPOUSE OR PARTNER							
205		How many wives do you have?	NO. OF WIVES:__ __<br>REFUSED TO SAY = 99	IF REFUSED → 301<br><br>MALES ONLY. SKIP FEMALE	C		
206		How many wives does your husband have?	NO. OF WIVES: __ __<br>DON'T KNOW=98<br>REFUSED TO SAY = 99	IF 1, DK, REFUSED → 210<br><br>FEMALES ONLY			
207		The Household Schedule listed [INSERT NUMBER OF REPORTED PARTNERS] household members as your wives/partners. Are all of the listed household members your wives/partners who live in the household?<br><br>Interviewer: confirm the number from the household roster	YES = 1<br>NO = 2<br>REFUSED TO SAY = 99	IF NO, REFUSED →209<br>MALES ONLY SKIP IF FEMALE	C		

NO.	VARNAME	QUESTIONS	CODING CATEGORIES	SKIPS/FILTERS	CORE/SUPPLEMENT	AKAIS ONLY	NOTES
		SCAN THE BARCODE OF THE WIFE FROM THE HOUSEHOLD LISTING					
208		Is [NAME] your wife/partner?<br><br>REPEAT FOR EACH WIFE/PARTNER	YES = 1<br>NO = 2<br>REFUSED TO SAY = 99	MALES ONLY SKIP IF FEMALE	C		
209		Does [NAME] live in the household?<br><br>REPEAT FOR EACH WIFE/PARTNER	YES = 1<br>NO = 2<br>REFUSED TO SAY = 99	MALES ONLY SKIP IF FEMALE	C		
210		Do you have additional spouse(s)/partner(s) that live with you?	YES = 1<br>NO = 2<br>REFUSED TO SAY = 99	IF NO, REFUSED →212<br>MALES ONLY SKIP IF FEMALE	C		
211		How many additional spouse(s)/partners(s) live with you?	NUMBER OF SPOUSES OR LIVE-IN PARTNERS _ _	MALES ONLY SKIP IF FEMALE	C		

NO.	VARNAME	QUESTIONS	CODING CATEGORIES	SKIPS/FILTERS	CORE/SUPPLEMENT	AKAIS ONLY	NOTES
212		What is the name of your spouse/partner that lives with you?	NAME OF SPOUSE/PARTNER ____<br>DON'T KNOW = 98<br>REFUSED = 99	MALES ONLY SKIP IF FEMALE	C		
213		Do you have additional spouse(s)/partner(s) that live elsewhere?	YES = 1<br>NO = 2<br>REFUSED TO SAY = 99	IF NO, REFUSED →301<br>MALES ONLY SKIP IF FEMALE		Y	
214		How many wives or live-in partners do you have who live elsewhere?	NUMBER OF ADDITIONAL SPOUSE(S)/PARTNERS ____<br>DON'T KNOW = 98<br>REFUSED = 99	MALES ONLY SKIP IF FEMALE<br><br>CANNOT BE >205	C		
215		Is your husband or partner living with you now or is he staying elsewhere?	LIVING TOGETHER = 1<br>STAYING ELSEWHERE = 2<br>DON'T KNOW = 98<br>REFUSE TO ANSWER = 99	IF STAYING ELSEWHERE, DK, REFUSED →218<br><br>IF LIVING TOGETHER & LISTED IN HH SCHEDULE	C		

NO.	VARNAME	QUESTIONS	CODING CATEGORIES	SKIPS/FILTERS	CORE/SUPPLEMENT	AKAIS ONLY	NOTES
				→215<br><br>FEMALES ONLY<br>SKIP IF MALE			
		SCAN THE BARCODE OF THE HUSBAND FROM THE HOUSEHOLD LISTING					
216		The household schedule listed [NAME OF HUSBAND/PARTNER] as your husband/partner who is living here. Is that correct?	YES=1<br>NO=2<br>DON'T KNOW = 98<br>REFUSED TO SAY = 99	IF YES, DK, REFUSED → 218<br>FEMALES ONLY<br>SKIP IF MALE	C		
217		Is your spouse/partner that lives with you on the HH roster?	LISTED ON THE HH ROSTER = 1<br>NOT LISTED IN HOUSEHOLD = 96	IF LISTED → 218<br>FEMALES ONLY<br>SKIP IF MALE	C		
		SCAN THE BARCODE OF THE SPOUSE/PARTNER FROM THE HOUSEHOLD LISTING					

NO.	VARNAME	QUESTIONS	CODING CATEGORIES	SKIPS/FILTERS	CORE/SUPPLEMENT	AKAIS ONLY	NOTES
218		What is the name of your spouse/partner that lives with you?	NAME OF SPOUSE/PARTNER ____<br>DON'T KNOW = 98<br>REFUSED = 99	FEMALES ONLY<br>SKIP IF MALE	C		
219		Does your husband or partner have other wives or does he live with other women as if married?	YES=1<br>NO=2<br>DON'T KNOW = 98<br>REFUSED TO SAY = 99	IF NO, DK, REFUSED<br>→301<br><br>FEMALES ONLY<br>SKIP IF MALE	C		
220		Including yourself, how many wives does your husband have?	NO. OF WIVES: __ __<br>DON'T KNOW=98<br>REFUSED TO SAY = 99	FEMALES ONLY<br>SKIP IF MALE	C		

MODULE 3: REPRODUCTIVE HEALTH

NO.	VARNAME	QUESTIONS	CODING CATEGORIES	SKIPS/FILTERS	CORE/SUPPLE-MENT	KADAIS ONLY	NOTES
**[INTERVIEWER READ]**: NOW I WOULD LIKE TO ASK YOU QUESTIONS ABOUT YOUR PREGNANCIES AND YOUR CHILDREN				IF MALE → 354			
301		How many times have you been pregnant? Include current pregnancy<br><br>CODE '00' IF NONE.	TIME(S) ____<br>NONE = 00<br>REFUSED TO SAY = 99	IF NONE, REFUSED → 354	C		
302		Have you ever had a pregnancy that resulted in a live birth?<br><br>A live birth is when the baby shows signs of life, such as breathing, beating of the heart or move-ment.	YES = 1<br>NO = 2<br>REFUSED TO SAY = 99	IF NO, REFUSED →354	C		
303		How many children have you given birth to in the last three years?<br><br>This includes babies that were born alive but later died.	NUMBER OF BABIES ____<br>DON'T KNOW = 98<br>REFUSED TO SAY = 99	IF ZERO, DK, RE-FUSED →354	C		

NO.	VARNAME	QUESTIONS	CODING CATEGORIES	SKIPS/FIL-TERS	CORE/SUPPLE-MENT	KADAIS ONLY	NOTES
NOW I WOULD LIKE TO ASK YOU SOME QUESTIONS ABOUT THE LAST PREGNANCY THAT RESULTED IN A LIVE BIRTH WITH IN THE LAST THREE YEARS.<br><br>SKIP TO 354 IF NO LIVE BIRTH IN THE LAST THREE YEARS.							
304		Did your last pregnancy result in birth to twins or more?	YES = 1<br>NO = 2<br>REFUSED TO SAY = 99				
305		What is the NAME of the baby/babies you delivered in your last pregnancy?<br><br>IF MULTIPLE BIRTH, LIST THE NAMES OF TWINS AND TRIPLETS. IF THE CHILD WAS NOT NAMED BEFORE DEATH, INPUT BIRTH 1 (BIRTH 2, BIRTH 3 FOR MULTI-PLE) ETC	INITIALS ______<br>INITIALS ______<br>INITIALS ______				
306		When did you give birth to **(NAME)**?	DATE:<br>DAY __ __<br>DON'T KNOW = 98<br>REFUSED TO SAY = 99<br><br>MONTH __ __<br>DON'T KNOW = 98<br>REFUSED = 99<br><br>YEAR __ __ __ __	MUST < 3 YEARS FROM TO-DAY'S DATE		Y	

			DON'T KNOW =9998<br>REFUSED TO SAY = 9999				
307		How old was **(NAME)** at his/her last birthday?<br>RECORD AGE IN YEARS<br>IF LESS THAN 1 YEAR, CODE '00'	YEARS __ __<br>DON'T KNOW = 98<br>REFUSED = 99	LESS THAN 4 YEARS			
308		Is **(INITIALS)** still alive?	YES = 1<br>NO = 2<br>REFUSED TO SAY = 99	REPEAT FOR MUL-TIPLES<br><br>IF YES→311	C		
309		How old was (NAME) when he/she died?	YEARS _____<br>DON'T KNOW = 98<br>REFUSED = 99	REPEAT FOR MULTI-PLES			
310		How old was (NAME) in months when he/she died?	MONTHS ______<br>DON'T KNOW = 98<br>REFUSED = 99	REPEAT FOR MULTI-PLES			
311		Is (**INITIALS**) a boy or a girl?	BOY(S)=1<br>GIRL(S)=2<br>REFUSED TO SAY = 99	REPEAT FOR MUL-TIPLES			

312		Is **(INITIALS)** living with you?	YES = 1<br>NO = 2<br>REFUSED = 99	REPEAT FOR MUL-TIPLES<br><br>ONLY IF BABY IS ALIVE FROM 308<br><br>IF NO→314			
313		SCAN THE BARCODE FROM THE HOUSEHOLD ROSTER					
314		Were you ever tested for HIV before your pregnancy with **(NAME)**?	YES = 1<br>NO = 2<br>DON'T KNOW = 98<br>REFUSED TO SAY = 99	IF NO, DK, REFUSED → 317			
315		Did you test positive for HIV before your pregnancy with **(NAME)**?	YES = 1<br>NO = 2<br>DON'T KNOW = 98<br>REFUSED TO SAY = 99	IF NO, DK, REFUSED → 317			
316		Were you taking ARVs or HIV medications, before you were pregnant with **(NAME)**?	YES = 1<br>NO = 2<br>DON'T KNOW = 98<br>REFUSED TO SAY = 99				

317		When you were pregnant with **(NAME)**, did you go to a health facility for antenatal care (ANC)?	YES = 1<br>NO = 2<br>DON'T KNOW = 98<br>REFUSED TO SAY = 99	IF YES<br>→319	C		
318		What was your <u>main</u> reason for not attending antena-tal care when you were pregnant with **(NAME)**?<br><br>SELECT ONLY ONE OPTION<br>PROBE FOR THE MAIN REASON	THE CLINIC IS TOO FAR AWAY = 1<br>COULD NOT TAKE TIME OFF WORK/TOO BUSY = 2<br>COULD NOT AFFORD TO PAY FOR THE VISIT = 3<br>DID NOT TRUST THE CLINIC STAFF = 4<br>RECEIVED CARE AT HOME = 5<br>DID NOT WANT AN HIV TEST DONE = 6<br>HUSBAND/FAMILY WOULD NOT LET ME GO = 7<br>USED TRADITIONAL BIRTH ATTENDANT = 8<br>POOR CLINICAL SERVICES =9<br>OTHER (SPECIFY)= 96<br>______________________________<br>DON'T KNOW = 98<br>REFUSED TO SAY = 99	→ 334	C		
319		At what month in your pregnancy did you start at-tending the antenatal clinic?	1-3 MONTHS/1ST TRIMESTER = 1<br>4-6 MONTHS/2ND TRIMESTER = 2<br>7-9 MONTHS/3RD TRIMESTER = 3<br>DON'T REMEMBER/DON'T KNOW = 98<br>REFUSED TO SAY = 99	USE AIDS TO HELP DEFINE TIME	S		
320		What type of clinic did you go for antenatal care when you were pregnant with **(NAME)**?	PUBLIC CLINIC/HOSPITAL= 1<br>PRIVATE CLINIC/HOSPITAL = 2		S		

			FAITH BASED CLINIC/HOSPITAL=3<br>DON'T KNOW = 98<br>REFUSED TO SAY = 99				
321		During your visits to the antenatal care clinic when you were pregnant with **(NAME),** were you offered HIV testing and counselling?	YES = 1<br>NO = 2<br>DON'T KNOW = 98<br>REFUSED TO SAY = 99	IF NO, DK, REFUSE<br>→323	C		
322		Were you tested for HIV during any of your antenatal clinic visits when you were pregnant with **(NAME)**?	YES = 1<br>NO = 2<br>DON'T KNOW = 98<br>REFUSED TO SAY = 99	IF YES<br>→324	C		
323		What was the main reason you were not tested for HIV during antenatal care/maternity services with **(NAME)**?<br><br>SELECT ONLY ONE OPTION<br>PROBE FOR MAIN REASON	DID NOT WANT AN HIV TEST DONE/DON'T WANT TO KNOW MY STATUS = 1<br>DID NOT RECEIVE PERMISSION FROM SPOUSE/FAMILY = 2<br>AFRAID OTHERS WILL KNOW ABOUT TEST RESULTS = 3<br>DON'T NEED TEST/LOW RISK = 4<br>TEST NOT DONE AT CLINIC=5<br>HIV POSITIVE=6<br>OTHER (SPECIFY) = 96<br>________________________________<br>DON'T KNOW = 98<br>REFUSED = 99	IF HIV POSITIVE<br>→327<br><br>ANYTHING ELSE<br>→330			
324		Did you receive the results?	YES = 1<br>NO = 2<br>DON'T KNOW = 98	IF NO, DK, REFUSED<br>→326	C-MODI-FIED		

			REFUSED TO SAY = 99				
325		What were the results of the last HIV test you received during your pregnancy with **(NAME)**?	POSITIVE = 1<br>NEGATIVE = 2<br>UNKNOWN = 3<br>DON'T KNOW = 98<br>REFUSED TO SAY = 99		C-MODIFIED		
326		Where were you tested for HIV during your pregnancy with **(NAME)**?<br><br>SELECT ALL THAT APPLY<br>PROBE FOR SPECIFIC TYPE OF SERVICE	ANC CLINIC = 1<br>LABOUR ROOM=2<br>LABORATORY=3<br>VOLUNTARY COUNSELING AND TESTING (VCT) CENTER = 4<br>OTHER (SPECIFY) = 96<br>_______________________________<br>DON'T KNOW = 98<br>REFUSED TO SAY = 99		S		
327		Did you get ARVs or HIV medications during your pregnancy to stop (**NAME**) from getting HIV?	YES = 1<br>NO = 2<br>DON'T KNOW = 98<br>REFUSED TO SAY = 99	HIV POSITIVE ONLY FROM 315 OR 325 (OPTION 1)<br><br>IF YES → 329	CC		

328		What was the main reason you did not get ARVs or HIV medicines for your own health while you were pregnant with **(NAME)**?<br><br>SELECT ONLY ONE OPTION<br>PROBE FOR MAIN REASON	HUSBAND/FAMILY DID NOT AGREE=1<br>HEALTH CARE PROVIDER DID NOT PRE-SCRIBE = 2<br>I FEEL HEALTHY/NOT SICK = 3<br>COST OF MEDICATIONS = 4<br>COST OF TRANSPORT = 5<br>RELIGIOUS REASONS = 6<br>TAKING TRADITIONAL MEDICATIONS = 7<br>I DIDN'T ACCEPT STATUS=8<br>OTHER (SPECIFY) =96<br>___________________________<br>DON'T KNOW = 98<br>REFUSED TO SAY = 99	→ 330	C		
329		At what month in your pregnancy were you when you started taking ARVs or HIV medicine?	MONTHS 1-3/1ST TRIMESTER = 1<br>MONTHS 4-6/2ND TRIMESTER = 2<br>MONTHS 7-9/3RD TRIMESTER = 3<br>DON'T KNOW = 98<br>REFUSED = 99		S-MODI-FIED		
SUBSECTION: SYPHILIS							
330		Were you offered a test for syphilis during your ANC visits for **(NAME)**?	YES = 1<br>NO = 2<br>DON'T KNOW = 98<br>REFUSED TO SAY = 99	IF NO, DK, REFUSED<br>→ 334	S		

331		Were you tested for syphilis during your ANC visits for **(NAME)**?	YES = 1<br>NO = 2<br>DON'T KNOW = 98<br>REFUSED TO SAY = 99	IF NO, DK, REFUSED → 334	S		
332		Did you test positive for syphilis during your preg-nancy with **(NAME)**?	YES = 1<br>NO = 2<br>DID NOT GET RESULT = 3<br>DON'T KNOW = 98<br>REFUSED TO SAY = 99	IF NO, DK, REFUSED → 334	S		
333		Did you get treatment for syphilis during your preg-nancy with **(NAME)**?	YES = 1<br>NO = 2<br>DON'T KNOW = 98<br>REFUSED TO SAY = 99		S		
SUBSECTION: TIME IN LABOR							
334		Where did you give birth to **(NAME)**?	AT A HEALTH FACILITY = 1<br>AT HOME = 2<br>AT CHURCH=3<br>OTHER (SPECIFY) = 96<br>______________________________<br>REFUSED TO SAY = 99.	IF HOME, CHURCH, OTHER, REFUSE →341	C		
335		Were you offered an HIV test during labor?	YES = 1<br>NO = 2<br>ALREADY POSITIVE = 3	IF NO, DK, REFUSED →341			

			DON'T KNOW = 98<br>REFUSED TO SAY = 99	IF AL-READY POSITIVE<br>→ 338			
336		Did you test for HIV during labor?	YES = 1<br>NO = 2<br>DON'T KNOW = 98<br>REFUSED TO SAY = 99	IF HIV POSITIVE FROM 315 OR 325 THEN SKIP TO<br>→ 339<br><br>IF NO, DON'T KNOW, REFUSED<br>→ 341			
337		What was the result of that test?	POSITIVE = 1<br>NEGATIVE = 2<br>UNKNOWN = 3<br>DID NOT RECEIVE RESULTS = 4<br>DON'T KNOW = 98<br>REFUSED TO SAY = 99	IF 2, 3, 4, 98,99<br>→341<br><br>IF HIV POSITIVE FROM 315 OR 325 THEN SKIP TO<br>→ 339			

338		During labor, were you offered ARVs to protect **(NAME)** from HIV?	YES = 1<br>NO = 2<br>DON'T KNOW = 98<br>REFUSED TO SAY = 99	IF NO, DK, RE-FUSED→ 341<br><br>ASK ONLY IF HIV POSITIVE FROM 315 OR 325 OR 337			
339		During labor, did you take ARVs or HIV medications?	YES = 1<br>NO, DID NOT TAKE = 2<br>NO, NOT OFFERED = 3<br>DON'T KNOW = 98<br>REFUSED TO SAY = 99	IF NO, DK, RE-FUSED→ 341<br><br>INCLUDE GRAPHIC OF ARVS.<br><br>IF HIV POSITIVE FROM 315 OR 325 OR 337	C		
340		Did you continue to take the ARVs or HIV medications after labor?	YES = 1<br>NO= 2<br>DON'T KNOW = 98<br>REFUSED TO SAY = 99	IF HIV POSITIVE FROM 315 OR 325 OR 337	C		

SUBSECTION: AFTER BIRTH					C		
341		Did **(NAME)** take any ARVs or HIV medications to stop him/her from getting HIV infection? This would be before **(NAME**'S) first HIV test.	YES = 1<br>NO = 2<br>DON'T KNOW = 98<br>REFUSED TO SAY = 99	ONLY IF HIV POSITIVE (FROM 315 OR 325 OR 337)<br><br>INSERT GRAPHIC OF ARVS.	C		
342		Did you ever breastfeed **(NAME)**?	YES = 1<br>NO = 2<br>REFUSED TO SAY = 99	IF NO, REFUSED → 346	C		
343		For how long did you breastfeed **(NAME)**?<br><br>ONLY ONE OPTION MAY BE SELECTED. FOR EXAMPLE, ANSWER ONLY IN WEEKS OR IN MONTHS.	WEEKS ____<br>MONTHS ____<br>STILL BREASTFEEDING=3<br>DON'T KNOW = 98<br>REFUSED = 99		C		
344		Did you continue taking ARVs while you were breastfeeding **(NAME)**?	YES = 1<br>NO = 2<br>REFUSED TO SAY = 99	FOR HIV POSITIVE ONLY (FROM 315 OR 325 OR 337)	S		

345		Did you stop taking ARVs or HIV medicines once you stopped breastfeeding?	YES = 1<br>NO = 2<br>DON'T KNOW = 8<br>REFUSED = 9	ONLY IF HIV POSI-TIVE (FROM 315 OR 325 OR 337) AND ON TREAT-MENT (341)		Y	
346		**FOR NON-BREASTFEEDING MOTHERS:**<br><br>After **(NAME)** was born, was he/she tested for HIV?<br><br>**FOR BREASTFEEDING MOTHERS:**<br><br>While you were breastfeeding, was **(NAME)** tested for HIV?	YES = 1<br>NO = 2<br>DON'T KNOW = 98<br>REFUSED TO SAY = 99	FROM 342<br><br>IF NO, DK, REFUSED →352<br><br>ONLY IF HIV POS-TIVE (FROM 315 OR 325 OR 337)		Y	
347		How old was **(NAME)** when he/she first tested for HIV?	WEEKS ____<br>MONTHS _____<br>YEARS _____<br>DON'T KNOW = 98	ONLY IF HIV POSI-TIVE (FROM		Y	

			REFUSED = 99	315 OR 325 OR 337)<br><br>CANNOT BE MORE THAN BREAST-FEEDING MONTHS (343)			
348		What was the result of **(NAME)**'s first HIV test?	POSITIVE, **(NAME)** HAS HIV = 1<br>NEGATIVE, **(NAME)** DOES NOT HAVE HIV = 2<br>UNKNOWN = 3<br>DID NOT RECEIVE RESULTS = 4<br>DON'T KNOW = 98<br>REFUSED TO SAY = 99	ONLY IF HIV POSI-TIVE (FROM 315 OR 325 OR 337)		Y	
349		After you stopped breastfeeding, was **(NAME)** tested for HIV?<br><br>SKIP IF NOT BREASTFED (FROM 342 OPTION 2)	YES = 1<br>NO = 2<br>DON'T KNOW = 98<br>REFUSED TO SAY = 99	ONLY IF HIV POSI-TIVE (FROM 315 OR 325 OR 337)<br><br>IF NO, DK, REFUSED → 351		Y	

350		What was the result of **(NAME)**'s HIV test?	POSITIVE, **(NAME)** HAS HIV = 1<br>NEGATIVE, **(NAME)** DOES NOT HAVE HIV = 2<br>UNKNOWN = 3<br>DID NOT RECEIVE RESULTS = 4<br>DON'T KNOW = 98<br>REFUSED TO SAY = 99	ONLY IF MOTHER-HIV POSI-TIVE (FROM 315 OR 325 OR 337)		Y	
351		**INTERVIEWER READ:** THANK YOU FOR THE INFOR-MATION REGARDING **(NAME).**					

NO.	VARNAME	QUESTIONS	CODING CATEGORIES	SKIPS/FILTERS	CORE/SUPPLE-MENT	AKAIS ONLY	NOTES
352		Are you pregnant now?	YES = 1<br>NO = 2<br>DON'T KNOW = 98<br>REFUSED TO SAY = 99	IF NO, DK, RE-FUSED 354	C		
353		How many months pregnant are you?	MONTHS _ _<br><br>DON'T KNOW = 98<br>REFUSED TO SAY = 99	SKIP TO 401	S		
**FAMILY PLANNING**							

NO.	VARNAME	QUESTIONS	CODING CATEGORIES	SKIPS/FILTERS	CORE/SUPPLE-MENT	AKAIS ONLY	NOTES
354		Are you (your partner) **currently** doing something or using any method to delay or avoid getting pregnant?	YES = 1<br>NO = 2<br>DON'T KNOW = 98<br>REFUSED TO SAY = 99	IF YES THEN 356	C		
355		Why are you not using a method to prevent pregnancy?<br><br>PROBE FOR ALL RESPONSES<br><br>RECORD ALL MENTIONED	NOT MARRIED/NO PARTNER = 1<br>NOT HAVING SEX = 2<br>INFREQUENT SEX = 3<br>MENOPAUSAL/HYSTERECTOMY = 4<br>CAN'T GET PREGNANT = 5<br>NOT MENSTRUATED SINCE LAST BIRTH = 6<br>BREASTFEEDING = 7<br>UP TO GOD/FATALISTIC = 8<br>RESPONDENT OPPOSED = 9<br>HUSBAND/PARTNER OPPOSED = 10<br>RELIGION PROHIBITS= 11<br>KNOWS NO METHOD = 12<br>KNOWS NO SOURCE = 13<br>SIDE EFFECTS/HEALTH CONCERNS = 14<br>LACK OF ACCESS/TOO FAR = 15<br>COSTS TOO MUCH = 16<br>PREFERRED METHOD NOT AVAILABLE =17<br>NO METHOD AVAILABLE = 18<br>INCONVENIENT TO USE = 19<br>INTERFERES WITH BODY'S NORMAL PROCESSES = 20<br>WANTS MORE CHILDREN=21<br>OTHER (SPECIFY) = 96<br>______________________________	END MODULE	S		

NO.	VARNAME	QUESTIONS	CODING CATEGORIES	SKIPS/FILTERS	CORE/SUPPLE-MENT	AKAIS ONLY	NOTES
			DON'T KNOW = 98<br>REFUSED = 99				
356		Which method are you (your partner) using?<br><br>SELECT ALL THAT APPLY.	FEMALE STERILIZATION = 1<br>MALE STERILIZATION = 2<br>PILL = 3<br>IUD/ "COIL" = 4<br>INJECTIONS = 5<br>IMPLANT = 6<br>MALE CONDOM = 7<br>FEMALE CONDOM = 8<br>RHYTHM/NATURAL METHODS = 9<br>WITHDRAWAL = 10<br>NOT HAVING SEX/ABSTINENCE =11<br>OTHER (SPECIFY)= 96<br>_______________________________<br>DON'T KNOW = 98<br>REFUSED TO SAY = 99		C		
357		Would you like to have a/another child?	YES, HAVE (A/ANOTHER) CHILD = 1<br>NO MORE/NONE = 2<br>NO, (PARTNER) CANNOT GET PREGNANT = 3<br>UNDECIDED/DON'T KNOW = 8<br>REFUSED = 9	IF NO MORE/NONE, DK, REFUSED →401	S		

NO.	VARNAME	QUESTIONS	CODING CATEGORIES	SKIPS/FILTERS	CORE/SUPPLE-MENT	AKAIS ONLY	NOTES
358		How long would you like to wait before the birth of a/another child? Give your best estimate.<br><br>PROBE FOR ESTIMATE	MONTHS ___<br>YEARS ___<br>OTHER (SPECIFY) = 96<br>______________________________<br>DON'T KNOW = 98<br>REFUSED = 99	END MODULE	S		

MODULE 4: CHILDREN

NO.	VAR-NAME	QUESTIONS	CODING CATEGORIES	SKIPS/FILTERS	CORE/SUPPLEMENT	AKAIS ONLY	NOTES
THE HOUSEHOLD SCHEDULE NOTED THAT [**NAME OF PARTICIPANT**] WILL FILL OUT THE CHILDREN'S MODULE FOR [**NUMBER OF CHILDREN**].<br><br>I AM GOING TO ASK YOU A NUMBER OF QUESTIONS ABOUT YOUR CHILD/CHILDREN REGARDING THEIR HEALTH AND WHERE THEY GET THEIR HEALTH SERVICES. WE WILL ASK YOU ABOUT THESE CHILDREN:<br><br>LIST OF HOUSEHOLD MEMBERS FROM HOUSEHOLD SCHEDULE<br><br>[**LIST OF CHILDREN**]							
401		DO NOT READ:<br><br>CHECK HOUSEHOLD SCHEDULE TO GET NUMBER OF CHILDREN FOR THIS PARTICIPANT<br><br>IF NONE RECORD '00'	NUMBER OF CHILDREN __ __	IF 00 → NEXT SECTION	C		
402		SCAN THE BARCODE OF THE CHILD FROM THE HOUSEHOLD LISTING	_______________				PROGRAM TO DETERMINE HOW TO LINK TO HH SCHEDULE

403		Interviewer: Begin with the youngest child<br><br>What is your first youngest/second youngest/ and so on / oldest child's first name or nick-name?	_______________	START WITH THE YOUNG-EST CHILD			
404		How old was **(NAME)** at his/her last birthday?<br><br>ENTER '0' IF CHILD IS LESS THAN ONE-YEAR-OLD AT PRESENT.	YEARS ___<br>DON'T KNOW = 98<br>REFUSED = 99	>0, DK, REFUSED →406<br><br><0 THEN GO TO 405<br><br>AGE CANNOT BE GREATER THAN 14 YEARS.	C		
405		How old is **[NAME]** in months?	MONTHS ___<br>DON'T KNOW = 98<br>REFUSED = 99		C		

406		Is (**NAME**) a boy or girl?	BOY = 1<br>GIRL = 2<br>DON'T KNOW = 98<br>REFUSED = 99		C		
407		Is **[NAME]** enrolled in school?	YES = 1<br>NO, CURRENTLY NOT IN SCHOOL = 2<br>NO, TOO YOUNG TO BE IN SCHOOL = 3<br>DON'T KNOW = 98<br>REFUSED = 99	IF NO, TOO YOUNG, DK, REFUSED → 412			
408		Was **[NAME]** enrolled in school during the previous school year?	YES = 1<br>NO = 2<br>DON'T KNOW = 98<br>REFUSED = 99	IF NO, DK, REFUSED →412			
409		What year was **[NAME]** in during the previous school year?	YEAR: _____<br>DON'T KNOW = 98<br>REFUSED = 99				
410		What is the highest level of school [NAME] has attended: primary or secondary?	PRIMARY = 1<br>SECONDARY = 2<br>DON'T KNOW = 98<br>REFUSED = 99	IF DK, REFUSED → 412			

411		What year is **[NAME]** in now?	YEAR _____<br>DON'T KNOW = 98<br>REFUSED = 99				
412		Has (**NAME**) ever received a blood transfusion?	YES = 1<br>NO = 2<br>DON'T KNOW=98<br>REFUSED TO SAY=99			Y	
413		Is (**NAME**) circumcised?	YES = 1<br>NO = 2<br>DON'T KNOW=98<br>REFUSED TO SAY=99	IF NO, DK, REFUSED → 417<br><br>MALES ONLY.<br><br>SHOW PICTURE OF CIRCUMCISED PENIS.	C		
414		How old was (**NAME**) when he was circumcised?<br><br>ONLY ONE OPTION MAY BE SELECTED.<br>FOR EXAMPLE, ANSWER ONLY IN YEARS OR IN MONTHS.	CODE '00' IF LESS THAN ONE MONTH.<br>MONTHS ____ (LESS THAN 12 MONTHS)<br>YEARS ____<br>DON'T KNOW = 98<br>REFUSED = 99	MALES ONLY.	S		

415		Where was **(NAME)** circumcised?	AT HOME = 1<br>IN A PUBLIC CLINIC OR HEALTH FACILITY = 2<br>PRIVATE CLINIC OR HELATH FACILITY = 3<br>OTHER (SPECIFY) = 96<br>_______________________________<br>DON'T KNOW = 98<br>REFUSED TO SAY=99	MALES ONLY		Y	
416		Who circumcised (**NAME**)?	TRADITIONAL PRACTITIONER/CIRCUM-CISER/LOCAL BABA = 1<br>CLINICIAN=2<br>OTHER (SPECIFY) = 96<br>_______________________________<br>DON'T KNOW = 98<br>REFUSED TO SAY=99	MALES ONLY<br><br>SKIP TO 418	C		
417		Are you planning to circumcise (**NAME**) in the future?	YES = 1<br>NO = 2<br>DON'T KNOW = 98<br>REFUSED TO SAY=99	MALES ONLY	S		
418		Has (**NAME**) ever been tested for HIV?	YES = 1<br>NO = 2<br>DON'T KNOW = 98<br>REFUSED TO SAY=99	IF NO, DK, RE-FUSED →419<br><br>IF YES – 420	C		

419		Why has **[NAME]** never been tested for HIV?<br><br>SELECT ALL THAT APPLY.<br><br>PROBE FOR ALL ANSWERS	DON'T KNOW WHERE TO TEST = 1<br>TEST COSTS TOO MUCH = 2<br>TRANSPORT COSTS TOO MUCH = 3<br>TOO FAR AWAY = 4<br>AFRAID OTHERS WILL KNOW ABOUT TEST RESULTS = 5<br>DON'T NEED TEST/LOW RISK = 6<br>DID NOT RECEIVE PERMISSION FROM SPOUSE/FAMILY = 7<br>AFRAID SPOUSE/PARTNER/FAMILY WILL KNOW RESULTS = 8<br>DON'T WANT TO KNOW CHILD HAS HIV = 9<br>CANNOT GET TREATMENT FOR HIV = 10<br>TEST KITS NOT AVAILABLE = 11<br>RELIGIOUS REASONS = 12<br>OTHER = 13<br>SPECIFY: ____________________<br>DON'T KNOW = 98<br>REFUSED = 99	→443			
420		How many times has (**NAME**) been tested for HIV?	NUMBER OF TIMES _____<br>DON'T KNOW = 98<br>REFUSED TO SAY= 99	CAN'T BE ZERO		Y	
421		Where were the HIV test(s) done?<br><br>SELECT ALL THAT APPLY.	CHILD WELLFARE CLINIC-1<br>IN-PATIENT WARD = 2<br>OUTPATIENT CLINIC =3<br>TB CLINIC = 4<br>POST-NATAL CLINIC=5		S		

			OTHER (SPECIFY) = 96<br>______________________________<br>DON'T KNOW = 98<br>REFUSED TO SAY=99				
422		What month and year was [NAME]'s last HIV test done?<br><br>IF LESS THAN ONE MONTH, CODE '00".	MONTH ___ ___<br>DON'T KNOW MONTH = 98<br>REFUSED MONTH = 99<br><br>YEAR ___ ___ ___ ____<br>DON'T KNOW YEAR = 98<br>REFUSED YEAR = 99	DATE RE-STRAINTS	C		
423		What was (**NAME**)'s last HIV test result?	POSITIVE = 1<br>NEGATIVE = 2<br>INDETERMINATE = 3<br>DID NOT RECEIVE RESULTS = 4<br>DON'T KNOW = 98<br>REFUSED TO SAY=99	IF NEG, INDET, DID NOT RECEIVE, DK, RE-FUSED→ 443	C		
424		What was the month and year of **(NAME)**'s first HIV positive test result? Please give your best guess.<br><br>This will be the very first HIV positive test result that you have received.<br><br>PROBE TO VERIFY DATE.	MONTH ___ ___<br>DON'T KNOW MONTH = 98<br>REFUSED MONTH = 99<br><br>YEAR ___ ___ ___ ____<br>DON'T KNOW YEAR = 98<br>REFUSED YEAR = 99		C		

425		Has (**NAME**) ever received HIV care from a health care provider?	YES = 1<br>NO = 2<br>DON'T KNOW = 98<br>REFUSED TO SAY=99	IF YES → 427	C		
426		What is the <u>main</u> reason why (**NAME**) has never seen a health care provider for HIV care?<br><br>SELECT ONLY ONE RESPONSE	FACILITY IS TOO FAR AWAY = 1<br>I DON'T KNOW WHERE TO GET HIV CARE = 2<br>COST OF CARE = 3<br>COST OF TRANSPORT = 4<br>I DON'T THINK HE/SHE NEEDS IT, HE/SHE IS NOT SICK = 5<br>I FEAR PEOPLE WILL KNOW THAT HE/SHE HAS HIV IF I TAKE HIM/HER TO A CLINIC = 6<br>HE/SHE IS TAKING TRADITIONAL MEDICINE = 7<br>DON'T KNOW = 98<br>REFUSED TO SAY= 99	→443	C		
427		After learning of (**NAME**)'s HIV diagnosis, what month and year did (**NAME**) <u>first</u> see a health care provider for HIV care?	MONTH __________<br>DON'T KNOW MONTH = 98<br>REFUSED MONTH= 99<br>YEAR ____________<br>DON'T KNOW YEAR =9998<br>REFUSED = 9999				
428		What month and year did (**NAME**) <u>last</u> see a health care provider for HIV care?	MONTH __________<br>DON'T KNOW MONTH = 98<br>REFUSED MONTH= 99<br>YEAR ____________<br>DON'T KNOW YEAR =9998<br>REFUSED = 9999		C		

429		What is the main reason for (**NAME**) not seeing a health care provider for HIV medical care in the past 6 months?<br><br>SELECT ONLY ONE RESPONSE<br><br>PROBE FOR THE MAIN REASON	FACILITY IS TOO FAR AWAY = 1<br>COST OF CARE = 2<br>COST OF TRANSPORT =3<br>I DON'T THINK HE/SHE NEEDS IT, HE/SHE IS NOT SICK = 4<br>I FEAR PEOPLE WILL KNOW THAT HE/SHE HAS HIV IF I TAKE HIM/HER TO A CLINIC = 5<br>RELIGIOUS REASONS = 6<br>HE/SHE IS TAKING TRADITIONAL MEDICINE = 7<br>OTHER (SPECIFY) = 96<br>______________________________<br>DON'T KNOW = 98<br>REFUSED = 99		C		
430		Has (**NAME**) ever had a CD4 count test?<br>The CD4 count tells you how sick you are with HIV and if you need take ARVs or HIV medications.	YES = 1<br>NO = 2<br>DON'T KNOW = 98<br>REFUSED TO SAY= 99	IF NO, DK, REFUSED→ 432	C		
431		What month and year did (**NAME**)'s health care provider last test his/her CD4 count?	MONTH __________<br>DON'T KNOW MONTH = 98<br>REFUSED MONTH= 99<br>YEAR ____________<br>DON'T KNOW YEAR =9998<br>REFUSED = 9999		C		
432		Has (**NAME**) ever taken ARVs or HIV medications, to treat his/her HIV infection?	YES = 1<br>NO = 2<br>DON'T KNOW = 98<br>REFUSED TO SAY= 99	IF YES → 434<br>USE GRAPHIC	C		

433		What is the <u>main</u> reason (**NAME**) has never taken ARVs or HIV medications?<br><br>SELECT ONLY ONE RESPONSE<br><br>PROBE FOR MAIN REASON	NOT ELIGIBLE FOR TREATMENT=1<br>HEALTH CARE PROVIDER DID NOT PRESCRIBE = 2<br>HIV MEDICINES NOT AVAILABLE = 3<br>I DON'T THINK NEEDS IT, HE/SHE IS NOT SICK = 4<br>COST OF MEDICATIONS = 5<br>COST OF TRANSPORT = 6<br>RELIGIOUS REASONS = 7<br>TAKING TRADITIONAL MEDICATIONS = 8<br>OTHER (SPECIFY) = 96<br>______________________________<br>DON'T KNOW = 98<br>REFUSED TO SAY= 99	→438	C		
434		When did (**NAME**) first start taking ARVs or HIV medications?	MONTH ___________<br>DON'T KNOW MONTH = 98<br>REFUSED MONTH= 99<br>YEAR _____________<br>DON'T KNOW YEAR =9998<br>REFUSED = 9999		C		
435		Is (**NAME**) currently taking ARVs or HIV medi-cations?	YES = 1<br>NO = 2<br>DON'T KNOW = 98<br>REFUSED TO SAY= 99	IF YES → 437	C		
436		What is the <u>main</u> reason why (**NAME**) is not taking ARVs or HIV medications?	I HAVE TROUBLE GIVING HIM/HER A TABLET EVERYDAY = 1<br>HAD SIDE EFFECTS/RASH = 2<br>FACILITY/PHARMACY TOO FAR AWAY TO GET MEDICATION REGULARLY = 3	→ 438	C		

			COST OF MEDICATIONS = 4<br>COST OF TRANSPORT = 5<br>HE/SHE IS HEALTHY; HE/SHE IS NOT SICK = 6<br>FACILITY WAS OUT OF STOCK = 7<br>RELIGIOUS REASONS=8<br>HE/SHE IS TAKING TRADITIONAL MEDICATIONS = 9<br>OTHER (SPECIFY) = 96<br>______________________________<br>DON'T KNOW = 98<br>REFUSED = 99				
437		People sometimes forget to take their ARVs. In the past 30 days, how many days has (**NAME**) missed taking any ARV pills (HIV medications)?	NUMBER OF DAYS ___ ___<br>DON'T KNOW = 98<br>REFUSED = 99		C		
438		Has (**NAME**) ever had a viral load test?<br><br>This is a test that measures how much HIV is in your blood.	YES= 1<br>NO= 2<br>DON'T KNOW = 98<br>REFUSED TO SAY= 99	If NO, DK, RE-FUSED →441	S		
439		What month and year was (**NAME**) <u>last</u> viral load test?	MONTH ___________<br>DON'T KNOW MONTH = 98<br>REFUSED MONTH= 99<br>YEAR _____________<br>DON'T KNOW YEAR =9998<br>REFUSED = 9999		S		

440		Were you told the result of (**NAME**)'s viral load test?	YES= 1<br>NO= 2<br>DON'T KNOW = 98<br>REFUSED TO SAY= 99		S		
441		Is (**NAME**) currently taking Septrin or Cotri-moxazole?	YES = 1<br>NO = 2<br>I DON'T KNOW WHAT IT IS = 3<br>DON'T KNOW = 98<br>REFUSED TO SAY= 99	IF YES (1), IDK (3), DK (98), RE-FUSED (99) → 443<br><br>SHOW GRAPHIC OF SEP-TRIN OR COTRI-MOXA-ZOLE.	C		
442		Can you tell me the <u>main</u> reason why (**NAME**) is not <u>currently</u> taking Septrin or cotrim daily?<br><br>SELECT ONLY ONE	NOT PRESCRIBED = 1<br>I HAVE TROUBLE GIVING HIM/HER A TABLET EVERYDAY = 2<br>HE/SHE HAD SIDE EFFECTS/RASH = 3<br>FACILITY/PHARMACY TOO FAR AWAY TO GET SEPTRIN OR COTROMOXIAZOLE REGULARLY = 4<br>HE/SHE DOES NOT NEED IT, HE/SHE IS NOT SICK = 5<br>PHARMACY/ FACILITY WAS OUT OF STOCK = 6<br>COST OF MEDICATIONS = 7		S		

			COST OF TRANSPORT = 8<br>DOCTOR SAID NO LONGER NEEDED = 9<br>OTHER (SPECIFY) = 96<br>_______________________________<br>DON'T KNOW = 98<br>REFUSED TO SAY = 99				
443		Has **[NAME]** ever visited a clinic for tuberculo-sis (TB) for diagnosis or treatment?	YES = 1<br>NO = 2<br>DON'T KNOW = -8<br>REFUSED = -9	NO, DK, REFUSED → SKIP TO END OF MOD-ULE	C		
444		Have you ever been told by a health care pro-vider that **[NAME]** had TB?	YES = 1<br>NO=2<br>DON'T KNOW = -8<br>REFUSED = -9	NO, DK, REFUSED → SKIP TO END OF MODULE	C		
445		Was **[NAME]** ever treated for TB?	YES = 1<br>NO = 2<br>DON'T KNOW = -8<br>REFUSED = -9	NO, DK, REFUSED → SKIP TO END OF MOD-ULE	C		

446		Is **[NAME]** currently on treatment for TB?	YES = 1<br>NO = 2<br>DON'T KNOW = 8<br>REFUSED = 9	NO, DK, REFUSED → 448	C		
447		The last time **[NAME]** was treated for TB, did [NAME] complete at least 6 months of treatment?	YES = 1<br>NO = 2<br>DON'T KNOW = 8<br>REFUSED = 9		C		
448		Did you ever receive a basic care kit that may have contained items for your HIV care?<br><br>These items may have included a bucket, a mosquito net, water guard, a filter cloth, and educational materials.	YES = 1<br>NO=2<br>DON'T KNOW = 98<br>REFUSED TO SAY= 99		S		
449		Thank you for the information about **[NAME]**.<br><br>DOES THE RESPONDENT HAVE ANOTHER CHILD AGED 0-14 YEARS?	YES = 1<br>NO = 2	YES→RE-TURN BE-GINNING OF MOD-ULE	C		
**INTERVIEWER: IF PARENT/GUARDIAN HAS ANOTHER CHILD UNDER 14 YEARS BESIDES (NAME), GO TO TOP, 401, AND ASK ABOUT NEXT YOUNGEST CHILD. IF NO OTHER CHILDREN, THEN CONTINUE TO NEXT SECTION. ASK EACH QUESTION FOR ALL CHILDERN AGE 0 MONTHS- 14 YEARS**							

MODULE 5: SEXUAL ACTIVITY

NO.	VARNAME	QUESTIONS	CODING CATEGORIES	SKIPS/FILTERS	CORE/SUPPLEMENT	AKAIS ONLY	NOTES
Now I would like to ask you some questions about your sexual activity in order to gain a better understanding of some important life issues. Let me assure you again that your answers are completely confidential and will not be told to anyone. If we should come to any question that you don't want to answer, just let me know and we will go to the next question.							
501		Have you ever had sex?	YES = 1<br>NO = 2<br>DON'T KNOW = 98<br>REFUSED TO SAY = 99	ONLY IF SINGLE, NEVER PREGNANT OR NEVER HAD CHILDREN<br><br>IF NO → 601		Y	
502		Have you ever had vaginal sex? This is where a man puts his penis into a woman's vagina.	YES = 1<br>NO = 2<br>DON'T KNOW = 98<br>REFUSED TO SAY = 99	IF NO → 505		Y	TRACKS TO CORE 601

NO.	VARNAME	QUESTIONS	CODING CATEGORIES	SKIPS/FILTERS	CORE/SUPPLEMENT	AKAIS ONLY	NOTES
503		At what age did you first have vaginal sex?	AGE IN YEARS _ _<br>DON'T KNOW = 98<br>REFUSED TO SAY = 99			Y	TRACKS TO CORE 601
504		Did you use a condom the first time you had vaginal sex?	YES = 1<br>NO = 2<br>DON'T KNOW = 98<br>REFUSED TO SAY = 99		S		
505		Some men and women like to have anal sex. This is where a man puts his penis in someone's anus. Have you ever practiced this?	YES = 1<br>NO = 2<br>DON'T KNOW = 98<br>REFUSED TO SAY = 99	IF NO & 502=1 CHECK 501 AND PROBE IF EVER HAD SEX.<br><br>IF NO→510	S		
506		At what age did you first have anal sex?	AGE IN YEARS __ __<br>DON'T KNOW = 98<br>REFUSED TO SAY = 99		S		

NO.	VARNAME	QUESTIONS	CODING CATEGORIES	SKIPS/FILTERS	CORE/SUPPLEMENT	AKAIS ONLY	NOTES
507		The first time you had anal sex, was a condom used?	YES = 1<br>NO = 2<br>DON'T KNOW = 98<br>REFUSED TO SAY = 99		S		
508		Have you had anal sex in the last 12 months?	YES = 1<br>NO = 2<br>DON'T KNOW = 98<br>REFUSED TO SAY = 99			Y	
509		How many different people have you had anal sex in the last 12 months?<br><br>IF NON-NUMERIC ANSWER: PROBE TO GET AN ESTIMATE.<br><br>IF NUMBER OF PARTNERS IS GREATER THAN 95, WRITE ' 95'.	NUMBER OF PARTNERS IN LAST 12 MONTHS __ __<br><br>DON'T KNOW = 98<br>REFUSED TO SAY= 99			Y	
510		How many different people have you had sex with in your lifetime?<br><br>IF NON-NUMERIC ANSWER: PROBE TO GET AN ESTIMATE.<br><br>IF NUMBER OF PARTNERS IS GREATER THAN 100, WRITE ' 100'.	NUMBER OF PARTNERS IN LIFETIME __ __ __<br>DON'T KNOW = 998<br>REFUSED TO SAY= 999		S		

NO.	VARNAME	QUESTIONS	CODING CATEGORIES	SKIPS/FILTERS	CORE/SUPPLEMENT	AKAIS ONLY	NOTES
IF LESS THAN 3 PARTNERS:** Now I would like to ask you some questions about the partners you have had sex with in the last 12 months.*<br>IF 3 OR GREATER:** Now I would like to ask you some questions about the LAST 3 partners you have had sex with in the past 12 months.*<br>***TO ALL**: Let me assure you again that your answers are completely confidential and will not be told to anyone.*<br><br>REPEAT FOR THE 3 MOST RECENT PARTNERS.							
511		I would like to ask you for initials of your last partners in last 12 months so I can keep track. You don't have to give me exact initials. First give me the initials of the last person you had sex with in the last 12 months.	INITIALS<br><br>____ ____				
512		Does **(INITIALS)** live in this household?	YES = 1<br>NO = 2<br>REFUSED TO SAY = 99	If NO →514			
513		SCAN THE BARCODE FROM THE HOUSEHOLD SCHEDULE OF **(INITIALS)**<br><br>IF THE PERSON IS NOT LISTED IN THE HOUSEHOLD, RECORD '00'.					

NO.	VARNAME	QUESTIONS	CODING CATEGORIES	SKIPS/FILTERS	CORE/SUPPLEMENT	AKAIS ONLY	NOTES
514		What is your relationship with **(INITIALS)**?	HUSBAND/WIFE = 1<br>LIVE-IN PARTNER = 2<br>PARTNER, NOT LIVING WITH RESPONDENT = 3<br>FRIEND/ACQUAINTANCE = 4<br>SEX WORKER = 5<br>SEX WORKER CLIENT =6<br>OTHER = 96<br>DON'T KNOW = 98<br>REFUSED TO SAY = 99		C		
515		What is the sex of this person?	MALE = 1<br>FEMALE = 2<br>DON'T KNOW = 98<br>REFUSED TO SAY = 99		C		
516		How long has it been since you first had sex with **(INITIALS)**?<br><br>IF LESS THAN ONE WEEK RECORD IN DAYS, IF LESS THAN ONE MONTH, RECORD IN WEEKS, OTHERWISE RECORD IN MONTHS.	DAYS:__ __<br>WEEKS:__ __<br>MONTHS:__ __<br>YEARS:__ __<br><br>DON'T KNOW = 98<br>REFUSED TO SAY = 99		S		
517		How old is this person? Please give your best guess.	YEARS ____<br>DON'T KNOW =98<br>REFUSED TO SAY = 99		C		

NO.	VARNAME	QUESTIONS	CODING CATEGORIES	SKIPS/FILTERS	CORE/SUPPLEMENT	AKAIS ONLY	NOTES
518		The last time you had sex with **(INITIALS)**, did you have oral sex, vaginal sex, or anal sex?<br><br>TICK ALL THAT APPLY<br><br>PROBE FOR ALL RESPONSES	ORAL=1<br>VAGINAL = 2<br>ANAL = 3<br>DON'T KNOW =98<br>REFUSED TO SAY = 99		S		
519		Did you use a condom the last time you had sex with (**INITIALS)**?	YES = 1<br>NO = 2<br>DON'T KNOW = 98<br>REFUSED TO SAY = 99		C		
520		In the last 12 months, did you have vaginal sex with **(INITIALS)** without using a condom?	YES = 1<br>NO = 2<br>NO VAGINAL SEX IN THE LAST 12 MONTHS = 3<br>DON'T KNOW = 98<br>REFUSED TO SAY = 99	SKIP IF NEVER HAD VAGINAL SEX (502)<br><br>SKIP IF REPONDENT IS MALE AND PARTNER IS MALE		Y	

NO.	VARNAME	QUESTIONS	CODING CATEGORIES	SKIPS/FILTERS	CORE/SUPPLEMENT	AKAIS ONLY	NOTES
521		In the last 12 months, did you have anal sex with **(INITIALS)** without using a condom?	YES = 1<br>NO = 2<br>NO ANAL SEX IN THE LAST 12 MONTHS = 3<br>DON'T KNOW = 98<br>REFUSED TO SAY = 99	SKIP IF NEVER HAD ANAL SEX		Y	
522		In the last 12 months, did you ever use a condom when you had sex with **(INITIALS)**?	YES = 1<br>NO = 2<br>DON'T KNOW = 98<br>REFUSED TO SAY = 99	IF NO, DK, REFUSED ➔524	S		
523		In the last 12 months, when you had sex with **(INITIALS)**, did the condom you were using ever break, leak or slip off during sex?	YES = 1<br>NO = 2<br>DON'T KNOW = 98<br>REFUSED TO SAY = 99		S		
524		Did you have sex with **(INITIALS)** because they provided you with material support?<br><br>Material support means helping you to pay for things, or giving you gifts or money, or opportunity.	YES = 1<br>NO = 2<br>DON'T KNOW = 98<br>REFUSED TO SAY = 99	SKIP IF SPOUSE<br><br>IF NO, DK, REFUSED →526	C		

NO.	VARNAME	QUESTIONS	CODING CATEGORIES	SKIPS/FILTERS	CORE/SUPPLEMENT	AKAIS ONLY	NOTES
525		In the last 12 months, what did you receive when you had sex with **(INITIALS)**?<br><br>SELECT ALL THAT APPLY.<br><br>PROBE FOR ALL RESPONSES	MONEY = 1<br>FOOD = 2<br>GOOD GRADES = 3<br>SCHOOL FEES = 4<br>EMPLOYMENT = 5<br>GIFTS/FAVORS = 6<br>TRANSPORT = 7<br>SHELTER/RENT = 8<br>PROTECTION = 9<br>OTHER (SPECIFY) = 96<br>______________<br>DON'T KNOW = 98<br>REFUSED TO SAY = 99		C		
526		Do you expect to have sex with **(INITIALS)** again?	YES =1<br>NO =2<br>DON'T KNOW = 98<br>REFUSED TO SAY = 99		C		
527		Does **(INITIALS)** know your HIV status?<br><br>HIV status could mean you are HIV negative or HIV positive.	YES =1<br>NO =2<br>DON'T KNOW = 98<br>REFUSED TO SAY = 99		C		

NO.	VARNAME	QUESTIONS	CODING CATEGORIES	SKIPS/FILTERS	CORE/SUPPLEMENT	AKAIS ONLY	NOTES
528		Do you know **(INITIALS)** HIV Status?<br><br>HIV status could mean you are HIV negative or HIV positive	YES =1<br>NO =2<br>REFUSED TO SAY = 99	IF NO, REFUSED ➔530		Y	
529		What is the HIV status of **(INITIALS)**?<br><br>READ REPSONSE ALOUD	I THINK **(INITIALS)** IS POSITIVE = 1<br>**(INTIALS)** TOLD ME HE/SHE IS POSITIVE = 2<br>**(INITIALS)** IS POSITIVE, TESTED TOGETHER = 3<br>I THINK **(INITIALS)** IS NEGATIVE = 4<br>**(INITIALS)** TOLD ME HE/SHE IS NEGATIVE = 5<br>**(INITIALS)** IS NEGATIVE, TESTED TOGETHER=6<br>DON'T KNOW STATUS = 98<br>REFUSED TO SAY= 99		C		
530		DOES THE RESPONDENT HAVE ANOTHER PARTNER IN THE LAST 12 MONTHS?<br><br>I will now ask you about the person you have had sex with prior to **(INITALS)**.	YES=1<br>NO=1	IF YES, REPEAT MODULE	C		

MODULE 6: HIV/AIDS KNOWLEDGE AND ATTITUDES

NO.	VARNAME	QUESTIONS	CODING CATEGORIES	SKIPS/FILTERS	CORE/SUPPLEMENT/OPTIONAL	AKAIS ONLY	NOTES
601		Can the risk of HIV transmission be reduced by having sex with only one uninfected partner who has no other partners?	YES = 1<br>NO = 2<br>DON'T KNOW = 98<br>REFUSED TO ANSWER=99		O		
602		Can people reduce their risk of getting HIV by using a condom every time they have sex?	YES = 1<br>NO = 2<br>DON'T KNOW = 98<br>REFUSED TO SAY=99		O		
603		Can people get HIV from mosquito bites?	YES = 1<br>NO = 2<br>DON'T KNOW = 98<br>REFUSED TO SAY=99		O		
604		Can people get HIV by sharing food with a person who has HIV?	YES = 1<br>NO = 2<br>DON'T KNOW = 98<br>REFUSED TO SAY=99		O		

NO.	VARNAME	QUESTIONS	CODING CATEGORIES	SKIPS/FILTERS	CORE/SUPPLEMENT/OPTIONAL	AKAIS ONLY	NOTES
605		Can people get HIV because of witchcraft or other supernatural means?	YES = 1<br>NO = 2<br>DON'T KNOW = 98<br>REFUSED TO SAY=99			Y	
606		Can a healthy-looking person have HIV?	YES = 1<br>NO = 2<br>DON'T KNOW = 98<br>REFUSED TO SAY=99		O		
607		Would you buy fresh vegetables from a shopkeeper or vendor if you knew that this person had HIV?	YES= 1<br>NO = 2<br>DON'T KNOW = 98<br>REFUSED TO SAY=99				
608		Do you think children living with HIV should be allowed to attend school with children who do not have HIV?	YES = 1<br>NO = 2<br>DON'T KNOW/NOT SURE/DEPENDS = 98<br>REFUSED = 99				
609		Do you think people hesitate to take an HIV test because they are afraid of how other people will react if the test result is positive for HIV?	YES = 1<br>NO = 2<br>DON'T KNOW/NOT SURE/DEPENDS = 98<br>REFUSED = 99				

NO.	VARNAME	QUESTIONS	CODING CATEGORIES	SKIPS/FILTERS	CORE/SUPPLEMENT/OPTIONAL	AKAIS ONLY	NOTES
610		Do people talk badly about people who are living with HIV, or who are thought to be living with HIV?	YES = 1<br>NO = 2<br>DON'T KNOW/NOT SURE/DEPENDS = 98<br>REFUSED = 99				
611		Do people living with HIV, or thought to be living with HIV, lose the respect of other people?	YES = 1<br>NO = 2<br>DON'T KNOW/NOT SURE/DEPENDS = 98<br>REFUSED = 99				
612		Do you fear that you could get HIV if you come into contact with the saliva of a person living with HIV?	YES = 1<br>NO =2<br>ALREADY HAS HIV = 3<br>DON'T KNOW/NOT SURE/DEPENDS = 98<br>REFUSED = 99				
613		Would you be ashamed if someone in your family had HIV?	YES= 1<br>NO = 2<br>DON'T KNOW = 98<br>REFUSED TO SAY=99				

MODULE 7: HIV/AIDS TESTING

NO.	VARNAME	QUESTIONS	CODING CATEGORIES	SKIPS/FILTERS	CORE/SUPPLEMENT/OPTIONAL	AKAIS ONLY	NOTES
701		Has a health care worker or outreach worker ever talked to you about HIV?	YES= 1<br>NO = 2<br>DON'T KNOW = 98<br>REFUSE TO SAY=99	IF NO, DK, REFUSED ➔703	S		
702		When was the last time a health care worker or outreach worker talked to you about HIV?	IN THE LAST 30 DAYS =1<br>IN THE LAST 3 MONTHS= 2<br>IN THE LAST YEAR =3<br>LONGER THAN A YEAR AGO =4<br>DON'T KNOW = 98<br>REFUSE TO SAY=99		S		
703		Have you <u>ever</u> been **tested** for HIV?	YES = 1<br>NO = 2<br>DON'T' KNOW =98<br>REFUSE TO SAY=99	IF YES➔705	C		
704		Why have you never been tested for HIV?<br><br>PROBE: Any other reason?	NO KNOWLEDGE ABOUT HIV TEST =1<br>DON'T KNOW WHERE TO GET ONE = 2<br>TEST COSTS TOO MUCH = 3<br>TRANSPORT TO VCT SITE TOO	→715	C		

NO.	VARNAME	QUESTIONS	CODING CATEGORIES	SKIPS/FILTERS	CORE/SUPPLEMENT/OPTIONAL	AKAIS ONLY	NOTES
		RECORD ALL MENTIONED.	MUCH = 4<br>VCT/HTC SITE TOO FAR AWAY =5<br>AFRAID OTHERS WILL KNOW ABOUT TEST/TEST RESULTS = 6<br>DON'T NEED TEST/LOW RISK = 7<br>AFRAID TO KNOW IF I HAVE HIV =8<br>CAN'T GET TREATMENT IF HAVE HIV =9<br>NEVER BEEN OFFERED A TEST = 10<br>DID NOT RECEIVED PREMISSION FROM SPOUSE/FAMILY=11<br>OTHER (SPECIFY) = 96<br>______________________________<br>DON’T KNOW = 98<br>REFUSE TO SAY=99				
705		Why did you test for HIV?<br><br>PROBE FOR ALL POSSIBLE ANSWERS<br><br>RECORD ALL MENTIONED	HEALTH CARE OR OUTREACH OFFERED TEST=1<br>TESTED DURING ANTENATAL CLINIC VISIT=2<br>I JUST WANTED TO KNOW=3<br>FELT AT RISK OR SICK=4<br>GOT A NEW PARTNER=5<br>OTHER (SPECIFY) = 96<br>______________________________<br>DON’T KNOW =98<br>REFUSE TO SAY=99		S		

NO.	VARNAME	QUESTIONS	CODING CATEGORIES	SKIPS/FILTERS	CORE/SUPPLEMENT/OPTIONAL	AKAIS ONLY	NOTES
706		When was your last HIV test?	LESS THAN 3 MONTHS AGO =1<br>3-5 MONTHS AGO = 2<br>6-11 MONTHS AGO = 3<br>1-2 YEARS AGO = 4<br>MORE THAN 2 YEARS AGO = 5<br>DON'T KNOW= 98<br>REFUSED TO SAY=99		C-MODIFIED		
707		Where was the last test done?	VCT FACILITY = 1<br>MOBILE VCT =2<br>AT HOME = 3<br>HEALTH CLINIC / FACIITY =4<br>HOSPITAL OUT PATIENT CLINIC =5<br>TB CLINIC = 6<br>STI CLINIC = 7<br>HOSPITAL INPATIENT WARDS = 8<br>BLOOD DONATING CENTER = 9<br>FAMILY PLANNING CLINIC = 10<br>ANTENATAL CLINIC=11<br>OTHER (SPECIFY) = 96<br>______________________________<br>DON'T KNOW=98<br>REFUSED TO SAY=99		C		

NO.	VARNAME	QUESTIONS	CODING CATEGORIES	SKIPS/FILTERS	CORE/SUPPLEMENT/OPTIONAL	AKAIS ONLY	NOTES
708		What was the result of your last HIV test?	POSITIVE=1<br>NEGATIVE=2<br>UNCERTAIN/INDETERMINATE=3<br>DID NOT RECEIVE THE RESULT=4<br>DON'T KNOW=98<br>REFUSED TO SAY=99	If NEG, UNCERT, IND, NO RESULT, DK, REF→715	C		
709		What was the month and year of your first HIV positive test result? Please give your best guess.<br><br>This will be the very first HIV positive test result that you have received.<br><br>PROBE TO VERIFY DATE.	MONTH ___ ___<br>DON'T KNOW MONTH = 8<br>REFUSED MONTH = 9<br><br>YEAR ___ ___ ___ ____<br>DON'T KNOW YEAR = 98<br>REFUSED YEAR = 99		C		
710		Who have you told that you are HIV positive?<br><br>TICK ALL THAT APPLY.<br><br>PROBE FOR ALL ANSWERS	NO ONE =1<br>SPOUSE/SEX PARTNER =2<br>DOCTOR =3<br>FRIEND =4<br>FAMILY MEMBER= 5<br>OTHER (SPECIFY) = 96<br>______________________________<br>DON'T KNOW=98<br>REFUSED TO SAY=99		C		

NO.	VARNAME	QUESTIONS	CODING CATEGORIES	SKIPS/FILTERS	CORE/SUPPLEMENT/OPTIONAL	AKAIS ONLY	NOTES
711		Have you visited a health facility to see a doctor or health provider in the last 12 months?	YES = 1<br>NO = 2<br>DON'T KNOW = 98<br>REFUSED TO SAY=99	IF NO→713	S		
712		During any of your visits to the health facility in the last 12 months, did a health provider offer you an HIV test?	YES = 1<br>NO = 2<br>DON'T KNOW = 98<br>REFUSED = 99		S		
713		Have you ever tested yourself for HIV in private using a self-test kit?<br><br>With a self-test kit you can test yourself for HIV at home. There are instructions on how to interpret the results.	YES = 1<br>NO = 2<br>DON'T KNOW = 98<br>REFUSED TO SAY=99		S		
714		In your lifetime, how many total times have you been tested for HIV?<br><br>PROBE FOR BEST ESTIMATE	NUMBER OF TIMES TESTED FOR HIV<br>__ __<br><br>DON'T KNOW = 98<br>REFUSED TO SAY=99		S		
715		What do you think are your chances of getting HIV?<br><br>READ OUT ALL RESPONSES	NO RISK AT ALL = 1<br>SMALL = 2<br>MODERATE = 3<br>GREAT = 4	IF MODERATE/ GREAT→717		Y	

NO.	VARNAME	QUESTIONS	CODING CATEGORIES	SKIPS/FILTERS	CORE/SUPPLEMENT/OPTIONAL	AKAIS ONLY	NOTES
			I ALREADY HAVE HIV = 5<br>DON'T KNOW = 98<br>REFUSED TO SAY=99				
716		Why do you think you have no risk/a small chance of getting HIV?<br><br>PROBE FOR ALL POSSIBLE REASONS<br><br>RECORD ALL MENTIONED	NEVER HAD SEX = 1<br>NOT HAVING SEX ANYMORE = 2<br>USES CONDOMS = 3<br>HAS ONLY ONE PARTNER = 4<br>LIMITS NUMBER OF PARTNERS = 5<br>PARTNER HAS NO OTHER PARTNER = 6<br>OTHER (SPECIFY) = 96<br>________________________<br>DON'T KNOW = 98<br>REFUSED TO SAY=99	SKIP TO 801		Y	
717		Why do you think you have moderate/great risk of getting HIV?<br><br>PROBE FOR ALL POSSIBLE REASONS<br><br>RECORD ALL MENTIONED	DOES NOT USE CONDOMS.= 1<br>HAS MORE THAN ONE PARTNER=2<br>PARTNER HAS OTHER PARTNERS =3<br>HOMOSEXUAL CONTACTS=4<br>HAD BLOOD TRANSFUSIONS/ INJECTIONS=5<br>OTHER (SPECIFY) = 96<br>________________________<br>DON'T KNOW = 98<br>REFUSED TO SAY=99			Y	

MODULE 8: HIV STATUS, CARE AND TREATMENT

NO.	VARNAME	QUESTIONS	CODING CATEGORIES	SKIPS/FILTERS	CORE/SUP-PLE-MENT/OP-TIONAL	AKAIS ONLY	NOTES
Now I'm going to ask you more about your experience with HIV support, care, and treatment.				SKIP IF NOT HIV POSITIVE (FROM 708) TO 901			
801		After learning of your HIV diagnosis, have you ever received HIV medical care from a health care provider?	YES = 1<br>NO = 2<br>DON'T KNOW = 98<br>REFUSED TO SAY = 99	IF YES → 803		C	
802		What is the main reason why you have never seen a health care provider for HIV medical care?<br><br>SELECT ONLY ONE	THE FACILITY IS TOO FAR AWAY = 1<br>I DON'T KNOW WHERE TO GET HIV MEDICAL CARE = 2<br>COST OF CARE = 3<br>COST OF TRANSPORT = 4<br>I FEEL HEALTHY/NOT SICK = 5<br>I FEAR PEOPLE WILL KNOW THAT I HAVE HIV IF I GO TO A CLINIC = 6<br>I'M TAKING TRADITIONAL MEDICINE= 7<br>RELIGIOUS REASONS = 8<br>OTHER (SPECIFY) = 96<br>__________________________<br>DON'T KNOW = 98<br>REFUSED TO SAY = 99	→ 901		C	

NO.	VARNAME	QUESTIONS	CODING CATEGORIES	SKIPS/FILTERS	CORE/SUP-PLE-MENT/OP-TIONAL	AKAIS ONLY	NOTES
803		After learning your HIV diagnosis, what month and year did you <u>first</u> see a health care provider for HIV medical care?	MONTH ___ ___<br>DON'T KNOW =98<br>REFUSE TO SAY=99<br><br>YEAR ___ ___ ___ ____<br>DON'T KNOW=9998<br>REFUSED TO SAY=9999		C		
804		How many months or years has it been since you <u>last</u> saw a health care provider for HIV medical care?	MONTH ___ ___<br>DON'T KNOW MONTH = 98<br>REFUSED TO SAY= 99<br><br>YEAR ___ ___<br>DON'T KNOW YEAR =9998<br>REFUSED TO SAY = 9999	IF LAST MEDICAL VISIT ≥2 MONTHS,→806.	C		
805		Following your last appointment, what is the date of your next scheduled visit?	MONTH ___ ___<br>DON'T KNOW MONTH = 98<br>REFUSED TO SAY= 99<br><br>YEAR ___ ___ ___ ____<br>DON'T KNOW YEAR =9998<br>REFUSED TO SAY = 9999			Y	

NO.	VARNAME	QUESTIONS	CODING CATEGORIES	SKIPS/FILTERS	CORE/SUP-PLE-MENT/OP-TIONAL	AKAIS ONLY	NOTES
806		What is the main reason for not seeing a health care provider for HIV medical care since your last visit?<br><br>SELECT ONLY ONE	THE FACILITY IS TOO FAR AWAY = 1<br>I DON'T KNOW WHERE TO GET HIV MEDICAL CARE = 2<br>COST OF CARE = 3<br>COST OF TRANSPORT = 4<br>I FEEL HEALTHY/NOT SICK = 5<br>I FEAR PEOPLE WILL KNOW THAT I HAVE HIV IF I GO TO A CLINIC = 6<br>I'M TAKING TRADITIONAL MEDICINE= 7<br>RELIGIOUS REASONS = 8<br>OTHER (SPECIFY) = 96<br>_____________________________<br>DON'T KNOW = 98<br>REFUSED TO SAY= 99		C		
807		Approximately how long does it take you to travel from your home (or workplace) to see a health care provider?	LESS THAN ONE HOUR = 1<br>ONE TO TWO HOURS = 2<br>MORE THAN TWO HOURS = 3<br>DON'T KNOW = 98<br>REFUSED TO SAY= 99		S		

NO.	VARNAME	QUESTIONS	CODING CATEGORIES	SKIPS/FILTERS	CORE/SUP-PLE-MENT/OP-TIONAL	AKAIS ONLY	NOTES
808		Approximately how much does it cost to travel from your home (or workplace) to the clinic?	COST _______<br>DON'T KNOW = 98<br>REFUSED = 99	USE LOCAL CURRENCY (i.e. Nigerian Naira).	S		
809		Have you ever had a CD4 count test?<br><br>The CD4 count tells you how sick you are with HIV and if you need to take ARVs or other HIV medications.	YES = 1<br>NO = 2<br>DON'T KNOW = 98<br>REFUSED = 99	NO, DK, REFUSED → 811<br><br>NO, DK, REFUSED & NEVER IN HIV CARE (801) → SKIP TO END OF MODULE			
810		What month and year were you last tested for your CD4 count?	MONTH ___ ___<br>DON'T KNOW MONTH = 98<br>REFUSED MONTH = 99<br>YEAR ___ ___ ___ ____<br>DON'T KNOW YEAR = 98<br>REFUSED YEAR = 99	SKIP TO END OF MODULE IF NEVER IN HIV CARE (801).			
811		Have you <u>ever</u> taken ARVs, that is, antiretroviral medications or HIV medications, to treat HIV infection?	YES = 1<br>NO = 2<br>DON'T KNOW = 98<br>REFUSED TO SAY = 99	IF YES → 813	C		

NO.	VARNAME	QUESTIONS	CODING CATEGORIES	SKIPS/FILTERS	CORE/SUP-PLE-MENT/OP-TIONAL	AKAIS ONLY	NOTES
812		What is the main reason you have never taken ARVs or HIV medica-tions?<br><br>SELECT ONLY ONE	NOT ELIGIBLE FOR TREATMENT=1<br>HEALTH CARE PROVIDER DID NOT PRESCRIBE = 2<br>HIV MEDICINES NOT AVAILABLE = 3<br>I FEEL HEALTHY/NOT SICK = 4<br>COST OF MEDICATIONS = 5<br>COST OF TRANSPORT = 6<br>RELIGIOUS REASONS = 7<br>TAKING TRADITIONAL MEDICATIONS = 8<br>NOT ATTENDING HIV CLINIC = 9<br>OTHER (SPECIFY) = 96<br>______________________________<br>DON'T KNOW = 98<br>REFUSED TO SAY=99	→819	C		
813		When did you first start taking ARVs or HIV medications?	MONTH ___ ___<br>DON'T KNOW =98<br>REFUSE TO SAY=99<br><br>YEAR ___ ___ ___ ____<br>DON'T KNOW=9998<br>REFUSED TO SAY=9999		C		

NO.	VARNAME	QUESTIONS	CODING CATEGORIES	SKIPS/FILTERS	CORE/SUP-PLE-MENT/OP-TIONAL	AKAIS ONLY	NOTES
814		Are you currently taking ARVs, that is, antiretroviral medications or HIV medications?	YES = 1<br>NO=2<br>DON'T KNOW = 98<br>REFUSED TO SAY = 99	IF YES → 816	C		
815		Can you tell me the main reason why you are not taking ARVs, antiretroviral medications or HIV medications?<br><br>PROBE FOR THE **MAIN** REASON<br><br>SELECT ONLY ONE	I HAVE TROUBLE TAKING A TABLET EVERYDAY =1<br>I HAD SIDE EFFECTS =2<br>FACILITY TOO FAR AWAY FOR ME TO GET MEDICINE REGULARLY = 3<br>COST OF MEDICATIONS =4<br>COST OF TRANSPORT = 5<br>I FEEL HEALTHY/NOT SICK =6<br>FACILITY WAS OUT OF STOCK =7<br>RELIGIOUS REASONS =8<br>TAKING TRADITIONAL MEDICATIONS = 9<br>OTHER (SPECIFY) = 96<br>______________________________<br>DON' T KNOW = 98<br>REFUSED TO SAY=99	→ 819	C		
816		People sometimes forget to take their ARVs. In the past 30 days, how many days have you missed	DAYS ______<br>DON'T KNOW = 98<br>REFUSED TO SAY = 99		C		

NO.	VARNAME	QUESTIONS	CODING CATEGORIES	SKIPS/FILTERS	CORE/SUP-PLE-MENT/OP-TIONAL	AKAIS ONLY	NOTES
		taking any of your ARV pills (HIV medications)?					
817		In the past 7 days, how many days have you missed taking any of your ARV pills (HIV medications)?	DAYS ______<br>DON'T KNOW = 98<br>REFUSED TO SAY =9 9			Y	
818		Did you take all your ARV pills yes-terday?	YES = 1<br>NO = 2<br>DON'T KNOW = 98<br>REFUSED TO SAY =99			Y	
819		Did you ever have a viral load test?<br><br>This is a test that measures how much HIV is in your blood.	YES= 1<br>NO= 2<br>DON'T KNOW =98<br>REFUSED TO SAY =99	IF NO, DK, REFUSED → 822	S		
820		When did you last have a viral load test?	MONTH ___ ___<br>DON'T KNOW =98<br>REFUSE TO SAY=99<br><br>YEAR ___ ___ ___ ____<br>DON'T KNOW=9998<br>REFUSED TO SAY=9999		S		

NO.	VARNAME	QUESTIONS	CODING CATEGORIES	SKIPS/FILTERS	CORE/SUP-PLE-MENT/OP-TIONAL	AKAIS ONLY	NOTES
821		Were you told the result of your viral load test?	YES= 1<br>NO= 2<br>DON'T KNOW = 98<br>REFUSED TO SAY = 99		S		
822		Are you currently taking Septrin or cotrim?	YES = 1<br>NO=2<br>DON'T KNOW = 98<br>REFUSED TO SAY = 99	IF YES, DK, REFUSED → 824<br><br>SHOW GRAPHIC OF SEPTRIN OR COTRIMOXAZOLE.	S		
823		Can you tell me the main reason why you are not currently taking Septrin or cotrim?<br><br>SELECT ONLY ONE	NOT BEEN PRESCRIBED= 1<br>I HAVE TROUBLE TAKING A TABLET EVERYDAY = 2<br>I HAD SIDE EFFECTS/RASH = 3<br>FACILITY TOO FAR AWAY FOR ME TO GET SEPTRIN OR COTRIMOXAZOLE REGULARLY = 4<br>COST OF MEDICATIONS = 5<br>COST OF TRANSPORT = 6<br>FEEL HEALTHY/NOT SICK =<br>FACILITY WAS OUT OF STOCK = 7<br>DOCTOR SAID NO LONGER NEEDED = 8<br>OTHER (SPECIFY) = 96<br>______________________________		S		

NO.	VARNAME	QUESTIONS	CODING CATEGORIES	SKIPS/FILTERS	CORE/SUP-PLE-MENT/OP-TIONAL	AKAIS ONLY	NOTES
			DON' T KNOW = 98<br>REFUSED TO SAY = 99				
824		In the last 12 months, how often did a health care provider weigh you?	EVERY VISIT = 1<br>SOME VISITS = 2<br>NEVER = 3<br>DON'T KNOW = 98<br>REFUSED TO SAY = 99	IF NEVER, DK, RE-FUSED → 827	S		
825		In the last 12 months, were you told by your health care provider that you were underweight or had a low weight?	YES = 1<br>NO = 2<br>DON'T KNOW = 98<br>REFUSED TO SAY =9 9	IF NO, DK, REFUSED → 827	S		
826		Were you given a nutritional sup-plement or referred for a nutri-tional consult?	NO, NEVER GIVEN SUPPLEMENT/REFERRED = 1<br>YES, GIVEN SUPPLEMENT = 2<br>YES, REFERRED = 3<br>BOTH GIVEN SUPPLEMENT AND REFERRED = 4<br>DON'T KNOW = 98<br>REFUSED TO SAY =99		S		

NO.	VARNAME	QUESTIONS	CODING CATEGORIES	SKIPS/FILTERS	CORE/SUP-PLE-MENT/OP-TIONAL	AKAIS ONLY	NOTES
827		Have you ever attended a support group for HIV-positive people?	YES = 1<br>NO=2<br>DON'T KNOW = 98<br>REFUSED TO SAY=99	IF NO, DK, REFUSED → 900	S		
828		In the last 12 months, how many times did you attend a support group?	CODE 00 IF NONE<br>NUMBER OF TIMES ________<br>DON'T KNOW = 98<br>REFUSED TO SAY = 99				
829		Which of the following do you re-ceive from the support group re-lated to your HIV infection?<br><br>CHECK ALL THAT APPLY.	POSITIVE LIVING MESSAGES = 1<br>INFORMATION ABOUT HIV SERVICES = 2<br>REMINDED OF IMPORTANCE OF TAKING ARV REGULARLY = 3<br>REMINDED TO KEEP HIV APPOINTMENTS = 4<br>REFILLS OF ART MEDICATION = 5<br>HOME-BASED CARE = 6<br>PICKING UP ARV MEDICATIONS = 7<br>PSYCHOSOCIAL SUPPORT = 8<br>LIVELIHOOD/MATERIAL SUPPORT = 9<br>NOTHING = 10<br>DON'T KNOW = 98<br>REFUSED TO SAY = 99		S		

NO.	VARNAME	QUESTIONS	CODING CATEGORIES	SKIPS/FILTERS	CORE/SUP-PLE-MENT/OP-TIONAL	AKAIS ONLY	NOTES
830		At the last HIV care visit, were you asked if you had any of the following: cough, fever, night sweats, or weight loss?	YES = 1<br>NO = 2<br>DON'T KNOW = 98<br>REFUSED TO SAY = 99				
831		In the last 12 months, have you experienced the following: cough, fever, night sweats and weight loss?	YES = 1<br>NO = 2<br>DON'T KNOW = 98<br>REFUSED TO SAY = 99	IF NO, DK, REFUSED<br>→ NEXT MODULE			
832		The last time you experienced any of the four symptoms (cough, fever, night sweats, weight loss), were any of the following tests done to look for TB?<br><br>*A sputum test is when the patient has to cough and collect the sample in a cup.*<br><br>CHECK ALL THAT APPLY	CHEST X-RAY = 1<br>SPUTUM TEST = 2<br>NONE OF THESE = 3<br>DON'T KNOW = 98<br>REFUSED TO SAY = 99				

NO.	VARNAME	QUESTIONS	CODING CATEGORIES	SKIPS/FILTERS	CORE/SUP-PLE-MENT/OP-TIONAL	AKAIS ONLY	NOTES
833		In the last 12 months, have you ever been given Isoniazid (INH) to prevent developing TB?	YES = 1<br>NO = 2<br>DON'T KNOW = 98<br>REFUSED TO SAY = 99	IF NO,DK, REFUSED →NEXT MODULE<br><br>SHOW GRAPHIC OF ISONIAZID.			
834		How many months did you take INH?	MONTHS ____<br>CURRENTLY TAKING INH = 96<br>DON'T KNOW = 98<br>REFUSED TO SAY = 99				

MODULE 9: TUBERCULOSIS AND OTHER HEALTH ISSUES

Now I will ask you about tuberculosis or TB.							
900		Have you ever visited a clinic for TB diagnosis or treatment?	YES = 1<br>NO=2<br>DON'T KNOW = 98<br>REFUSED = 99	IF NO, DK, RE-FUSE→1001			
901		Have you ever been told by a doc-tor, clinical officer or nurse that you had TB?	YES = 1<br>NO=2<br>DON'T KNOW = 98<br>REFUSED = 99				
902		Were you ever treated for TB?	YES = 1<br>NO = 2<br>DON'T KNOW = 98<br>REFUSED = 99				
903		Are you currently on treatment for TB?	YES = 1<br>NO = 2<br>DON'T KNOW = 98<br>REFUSED = 99				

904		The last time you were treated for TB, did you complete at least 6 months of treatment?	YES = 1<br>NO =2<br>DON'T KNOW = 98<br>REFUSED = 99				

FOR FEMALE RESPONDENTS ONLY
Now I'm going to ask you about tests a health care provider can do to check for cervical cancer. The cervix connects the uterus to the vagina. The tests a health care provider can do to check for cervical cancer are called a Pap smear, HPV test and VIA test.

For a Pap smear and HPV test, a health care provider puts a small stick inside the vagina to wipe the cervix and sends the sample to the laboratory. For a VIA test, a healthcare worker puts vinegar on the cervix and looks to see if the cervix changes color.

905		Have you ever been tested for cer-vical cancer?	YES = 1<br>NO = 2<br>DON'T KNOW = 98<br>REFUSED TO SAY = 99	IF NO, DK, REFUSED → SKIP TO NEXT MODULE<br>FEMALE ONLY.	S		
906		When was your last test for cervi-cal cancer?	MONTH ___ ___<br>DON'T KNOW =98<br>REFUSE TO SAY=99<br><br>YEAR ___ ___ ___ ____<br>DON'T KNOW=9998<br>REFUSED TO SAY=9999	FEMALE ONLY	S		

907		What was the result of your last test for cervical cancer?	NORMAL = 1<br>ABNORMAL = 2<br>DON'T KNOW = 98<br>REFUSED TO SAY = 99	If 1 / 98/ 99 →NEXT MODULE<br><br>FEMALE ONLY	S		
908		Did you receive treatment after your last test for cervical cancer?	YES, I WAS TREATED ON THE SAME DAY = 1<br>YES, I RECEIVED TREATMENT ON A DIFFERENT DAY = 2<br>NO, DID NOT RECEIVE TREATMENT = 3<br>REFERRED = 4<br>DON'T KNOW = 98<br>REFUSED TO SAY = 99	FEMALE ONLY	S		

MODULE 10: BLOOD SAFETY AND MEDICAL INJECTIONS

NO.	VARNAME	QUESTIONS	CODING CATEGORIES	SKIPS/FILTERS	CORE/SUPPLEMENT/OPTIONAL	AKAIS ONLY	NOTES
1001		Have you ever had a blood transfusion?	YES = 1<br>NO = 2<br>DON'T KNOW = 98<br>REFUSED TO SAY=99	IF NO, DK, REFUSED→ 1003		Y	
1002		In what month and year was the last time you had a blood transfusion?	MONTH ___ ___<br>DON'T KNOW =98<br>REFUSE TO SAY=99<br><br>YEAR ___ ___ ___ ____<br>DON'T KNOW=9998<br>REFUSED TO SAY=9999			Y	
1003		Have you ever donated blood?	YES = 1<br>NO = 2<br>DON'T KNOW = 98<br>REFUSED TO SAY=99	IF NO, DK, REFUSED → 1011		Y	

NO.	VARNAME	QUESTIONS	CODING CATEGORIES	SKIPS/FILTERS	CORE/SUPPLEMENT/OPTIONAL	AKAIS ONLY	NOTES
1004		Have you donated blood in the last 12 months?	YES = 1<br>NO = 2<br>DON'T KNOW = 98<br>REFUSED TO SAY=99	IF NO, DK, REFUSED → 1010		Y	
1005		How many times did you donate blood in the last 12 months?	NUMBER OF TIMES: __ __<br><br>DON'T KNOW = 98<br>REFUSED TO SAY=99			Y	
1006		The last time you donated blood, were you asked to donate or did you donate voluntarily?	WAS ASKED TO DONATE = 1<br>DONATED VOLUNTARILY = 2<br>DON'T KNOW = 98<br>REFUSED TO SAY=99	IF 2, DK (98), REFUSED (99) → 1008		Y	
1007		Who asked you to donate blood the last time?	FAMILY / FRIENDS = 1<br>NATIONAL BLOOD TRANSFUSION SERVICE (NBTS) = 2<br>HOSPITAL = 3<br>OTHER (SPECIFY) = 96<br>______________________________<br>DON'T KNOW = 98<br>REFUSED TO SAY=99			Y	

NO.	VARNAME	QUESTIONS	CODING CATEGORIES	SKIPS/FILTERS	CORE/SUPPLEMENT/OPTIONAL	AKAIS ONLY	NOTES
1008		Where was your <u>last</u> blood donation made?	MOBILE DRIVE (SCHOOL, COLLEGE, CHURCH, WORKPLACE, PUBLIC GATHERING) = 1<br>NIGERIA NATIONAL TRANFUSION CENTER = 2<br>PUBLIC HOSPITAL = 3<br>MISSION HOSPITAL = 4<br>PRIVATE HOSPITAL = 5<br>OTHER (SPECIFY) = 96<br>_____________________________<br>DON'T KNOW = 98<br>REFUSED TO SAY=99			Y	
1009		What was the <u>main</u> reason you donated blood this <u>last time?</u><br><br>SELECT ONLY ONE	IN RESPONSE TO PUBLIC REQUESTS FOR BLOOD DONORS (CIVIC/ALTRUISTIC REASONS) = 1<br>AS PART OF A BLOOD COLLECTION DRIVE (THERE WAS A BLOOD DRIVE WHERE I WORK, GO TO SCHOOL, SHOP) = 2<br>SPECIFICALLY FOR A FAMILY MEMBER OR FRIEND = 3<br>AS PART OF A BLOOD DONOR CLUB = 4<br>IN EXCHANGE FOR COMPENSATION (FROM A FRIEND OR FAMILY) = 5<br>I AM A REGULAR DONOR = 6<br>TO KNOW MY HIV STATUS = 7			Y	

NO.	VARNAME	QUESTIONS	CODING CATEGORIES	SKIPS/FILTERS	CORE/SUPPLEMENT/OPTIONAL	AKAIS ONLY	NOTES
			OTHER (SPECIFY) = 96<br>______________________________<br>DON'T KNOW = 98<br>REFUSED TO SAY=99				
1010		Are you planning to donate blood in the future?	YES = 1<br>NO = 2<br>DON'T KNOW = 98<br>REFUSED TO SAY=99			Y	
1011		Now I would like to ask you some questions about any injections you have had in the last 12 months.<br>Have you had an injection for any reason in the <u>last 12 months</u>?	YES = 1<br>NO = 2<br>DON'T KNOW = 98<br>REFUSED TO SAY=99	IF NO, DK, REFUSED→ 1101 NEXT MODULE		Y	
1012		Have you had an injection in the <u>last</u> 12 months that was administered by a doctor, a clinical officer, a nurse, a pharmacist, a dentist, or any other health worker?	YES = 1<br>NO = 2<br>DON'T KNOW = 98<br>REFUSED TO SAY=99	IF NO, DK, REFUSED →1015		Y	

NO.	VARNAME	QUESTIONS	CODING CATEGORIES	SKIPS/FILTERS	CORE/SUPPLEMENT/OPTIONAL	AKAIS ONLY	NOTES
1013		How many injections did you have?<br><br>IF NUMBER OF INJECTIONS IS GREATER THAN 90, OR DAILY FOR 3 MONTHS OR MORE, RECORD '90'.<br><br>IF NON-NUMERIC ANSWER, PROBE TO GET AN ESTIMATE	NUMBER OF INJECTIONS:__ __<br><br>DON'T KNOW = 98<br>REFUSED TO DAY-99			Y	
1014		The last time you received an injection from a health worker, did the health worker take the syringe and needle from a new, unopened package?	YES = 1<br>NO=2<br>DON'T KNOW = 98<br>REFUSED TO SAY=99			Y	
1015		Have you had an injection in the last 12 months that was administered by a traditional practitioner or healer?	YES = 1<br>NO=2<br>DON'T KNOW = 98<br>REFUSED TO SAY=99	If NO, DK, REFUSED →1017		Y	
1016		In the last 12 months, have you given yourself an injection that was prescribed by a doctor, a clinical officer, a nurse, a pharmacist, a dentist, or any other health worker?	YES = 1<br>NO=2<br>DON'T KNOW = 98<br>REFUSED TO SAY=99			Y	

NO.	VARNAME	QUESTIONS	CODING CATEGORIES	SKIPS/FILTERS	CORE/SUPPLEMENT/OPTIONAL	AKAIS ONLY	NOTES
1017		If you have a choice, would you like to receive medication as an injection or pill?	INJECTION = 1<br>PILL = 2<br>NO PREFERENCE = 3<br>DON'T KNOW = 98<br>REFUSED TO SAY=99			Y	

MODULE 11: NON-PRESCRIPTION DRUG USE/ALCOHOL USE

NO.	VARNAME	QUESTIONS	CODING CATEGORIES	SKIPS/FILTERS	CORE/SUPPLEMENT/OPTIONAL	AKAIS ONLY	NOTES
1101		How often do you have a drink containing alcohol?	NEVER = 1<br>MONTHLY OR LESS = 2<br>2-4 TIMES A MONTH =3<br>2-3 TIMES A WEEK = 4<br>4 OR MORE TIMES A WEEK = 5<br>DON'T KNOW = 98<br>RESFUSED TO SAY=99	IF NEVER, DK, RESFUSED→1104			
1102		How many drinks containing alcohol do you have on a typical day when you are drinking?	1 OR 2= 1<br>3 OR 4= 2<br>5 OR 6 =3<br>7 TO 9 = 4<br>10 OR MORE = 5<br>DON'T KNOW = 98<br>RESFUSED TO SAY=99		O		
1103		On one occasion, how often do you have six or more drinks?	NEVER = 1<br>LESS THAN MONTHLY = 2<br>MONTHLY = 3<br>WEEKLY = 4<br>DAILY OR ALMOST DAILY = 5<br>DON'T KNOW = 98<br>RESFUSED TO SAY=99		O		

NO.	VARNAME	QUESTIONS	CODING CATEGORIES	SKIPS/FILTERS	CORE/SUPPLEMENT/OPTIONAL	AKAIS ONLY	NOTES
1104		In the past 12 months which of the following substances have you used to get high?<br><br>READ OUT ALOUD	COCAINE 1<br>HEROINE=2<br>INDIAN HEMP=3<br>TRANQUILIZERS=4<br>CODEINE=5<br>SNIFFING PETROL=6<br>SNIFFING TOBACCO=7<br>SNIFFING BURNING RUBBER=8<br>SNIFING GUM=9<br>INHALING SEWAGE/ GUTTERS=10<br>NEVER USED=11<br>OTHER (SPECIFY) = 96<br>______________________________<br>DON'T KNOW = 98<br>RESFUSED TO SAY=99			Y	
1105		Some people inject drugs with a needle and syringe for pleasure. Have you ever injected drugs for pleasure?	YES = 1<br>NO = 2<br>DON'T KNOW = 98<br>RESFUSED TO SAY=99	IF NO, DK, REGUSED END INTERVIEW	O		
1106		Have you injected drugs with a needle and syringe in the past 3 months?	YES = 1<br>NO = 2<br>DON'T KNOW = 98<br>RESFUSED TO SAY=99	IF NO, DK, REGUSED END INTERVIEW	O		

NO.	VARNAME	QUESTIONS	CODING CATEGORIES	SKIPS/FILTERS	CORE/SUPPLEMENT/OPTIONAL	AKAIS ONLY	NOTES
1107		When you have injected drugs during the last 3 months, have you shared the syringe or needle with other people?	YES = 1<br>NO = 2<br>DON'T KNOW = 98<br>RESFUSED TO SAY=99	IF NO, DK, REFUSED END INTERVIEW	O		
1108		Did you know the HIV status of everyone with whom you were sharing needles?	YES = 1<br>NO = 2<br>DON'T KNOW = 98<br>RESFUSED TO SAY=99	END INTERVIEW		Y	
