# Supplementary material for: Akwa Ibom AIDS indicator survey: Key findings and lessons learnt
Source: PLoS One. 2020 Jun 17;15(6):e0234079. doi: 10.1371/journal.pone.0234079 (PMC7299391; doi:10.1371/journal.pone.0234079)
Supplement: S1 Appendix — (ZIP) [file pone.0234079.s001.zip › Questionnaires/AKAIS Household Schedule.pdf]

AKAIS Household Schedule

LINE NO.	USUAL RESIDENTS AND VISITORS	RELATIONSHIP TO HEAD OF HOUSEHOLD	SEX	RESIDENCE		AGE	
1	2	3	4	5	6	7	8
	Please give me the first names of the persons who usually live in your household or guests of the household who stayed here last night, starting with the head of the household.<br><br>*A person who usually lives in your household is someone who regularly consumes or contributes to food and other shared household resources.*<br><br>*AFTER LISTING THE NAME AND RECORDING THE RELATIONSHIP AND SEX FOR EACH PERSON ASK QUESTIONS 2A-2C BELOW TO BE SURE THAT THE SCHEDULE IS COMPLETE.*	*What is the relationship of (**NAME**) to the head of the household?*<br><br>*SEE CODES BELOW*	*Is (**NAME**) Male or Female?*	*Does (**NAME**) usually live here?*	*Did (**NAME**) sleep here last night?*	*How old is (**NAME**)?*	*INDICATE IN MONTHS or YEARS*
1		☐☐	M F	Y N	Y N		MONTHS ☐<br>YEARS ☐
2		☐☐	M F	Y N	Y N		MONTHS ☐<br>YEARS ☐
3		☐☐	M F	Y N	Y N		MONTHS ☐<br>YEARS ☐
4		☐☐	M F	Y N	Y N		MONTHS ☐<br>YEARS ☐
5		☐☐	M F	Y N	Y N		MONTHS ☐<br>YEARS ☐
6		☐☐	M F	Y N	Y N		MONTHS ☐<br>YEARS ☐
7		☐☐	M F	Y N	Y N		MONTHS ☐<br>YEARS ☐
8		☐☐	M F	Y N	Y N		MONTHS ☐<br>YEARS ☐
9		☐☐	M F	Y N	Y N		MONTHS ☐<br>YEARS ☐
10		☐☐	M F	Y N	Y N		MONTHS ☐<br>YEARS ☐

CONFIDENTIAL

TICK HERE IF CONTINUATION SHEET USED	*YES* *NO*	***CODES FOR COLUMN 3: RELATIONSHIP TO HOUSEHOLD HEAD***
2A) *Just to make sure I have a complete listing, are there any other persons such as small children or infants that we have not listed?*	*YES* *NO*	*01 = HEAD*<br>*02 = WIFE/HUSBAND/PARTNER*<br>*03 = SON OR DAUGHTER*<br>*04 = SON-IN-LAW/ DAUGHTER-IN-LAW RELATIVE*<br>*05 = GRANDCHILD*<br>*06 = PARENT*<br>*07 = PARENT-IN-LAW*<br>*08 = BROTHER/SISTER*<br>*09 = NIECE/NEPHEW*<br>*10 = CO-PARTNER*<br>*11 = CO-WIFE*<br>*12 = OTHER*<br>*13 = ADOPTED/ FOSTER/STEPCHILD*<br>*14 = NOT RELATED*<br>*98 = DON'T KNOW*
2B) *Are there any other people who may not be members of your household such as domestic servants, lodgers, or friends who usually live here?*	*YES* *NO*	
2C) *Are there any guests or temporary visitors staying here, or anyone else who stayed here last night who we have not listed?*	*ADD TO SCHEDULE*	

TOTAL ELIGIBLE MEN (ADULTS AND MATURE MINORS)	
TOTAL ELIGIBLE WOMEN (ADULTS AND MATURE MINORS)	
TOTAL ELIGIBLE CHILDREN (10 TO 14 YEARS)	
TOTAL CHILDREN (0 MONTHS TO 9 YEARS)	

CONFIDENTIAL

CONFIDENTIAL

HOUSEHOLD SCHEDULE							
LINE NO.	IF (NAME) IS **0-17 YEARS**					IF (NAME) IS 0-14 YEARS	
	Mature Minor Status if NAME is 15-17 years	ORPHAN STATUS/PARENT OR GUARDIAN					
	Is (**NAME**) a mature minor?<br><br>[A mature minor is a person 15-17 years of age who is married, pregnant has children, or is no longer under the care of a parent/guardian]	Is (**NAME**)'s natural mother alive?	Does (**NAME**)'s natural mother usually live in this household or was a guest last night?<br><br>IF YES:<br>RECORD MOTHER'S LINE NUMBER.<br><br>IF NO:<br>RECORD **FEMALE** GUARDIAN'S LINE NUMBER OR '00' IF FEMALE PARENT OR GUARDIAN NOT PRESENT IN HH.	Is (**NAME**)'s natural father alive?	Does (**NAME**)'s natural father usually live in this household or was a guest last night?<br><br>IF YES:<br>RECORD FATHER'S LINE NUMBER.<br><br>IF NO:<br>RECORD **MALE** GUARDIAN'S LINE NUMBER OR '00' IF MALE PARENT OR GUARDIAN NOT PRESENT IN HH.	RECORD LINE NUMBER OF PARENT/GUARDIAN WHO WILL FILL OUT CHILDREN'S MODULE FOR **(NAME)**	DO NOT READ:<br><br>IS (**NAME**) ELIGIBLE FOR SURVEY?
(1)	(9)	(10)	(11)	(12)	(13)	(14)	(15)
1	Y N DK	Y N—DK<br>↓<br>12	☐☐	Y N—DK<br>↓<br>14	☐☐	☐☐	Y N
2	Y N DK	Y N—DK<br>↓<br>12	☐☐	Y N—DK<br>↓<br>14	☐☐	☐☐	Y N
3	Y N DK	Y N—DK<br>↓<br>12	☐☐	Y N—DK<br>↓<br>14	☐☐	☐☐	Y N
4	Y N DK	Y N—DK<br>↓<br>12	☐☐	Y N—DK<br>↓<br>14	☐☐	☐☐	Y N

5	Y N DK	Y N —DK ↓ 12		Y N —DK ↓ 14			Y N
6	Y N DK	Y N —DK ↓ 12		Y N —DK ↓ 14			Y N
7	Y N DK	Y N —DK ↓ 12		Y N —DK ↓ 14			Y N
8	Y N DK	Y N —DK ↓ 12		Y N —DK ↓ 14			Y N
9	Y N DK	Y N —DK ↓ 12		Y N —DK ↓ 14			Y N
10	Y N DK	Y N —DK ↓ 12		Y N —DK ↓ 14			Y N

CONFIDENTIAL

HOUSEHOLD SCHEDULE

	IF (NAME) is 18 years or older or mature minor (see 9-11)			
LINE NO.	WIVES AND CO-HABITATING PARTNERS			
(1)	23a	23b	23c	23d
	Record the LINE NUMBER of (**NAME**)'s wife or partner. If no wife or partner leave blank.	Record the LINE NUMBER of (**NAME**)'s wife or partner. If no wife or partner leave blank.	Record the LINE NUMBER of (**NAME**)'s wife or partner. If no wife or partner leave blank.	Record the LINE NUMBER (**NAME**)'s of wife or partner. If no wife or partner leave blank.
(1)	(27a)	(27b)	(27c)	(27d)
1				
2				
3				
4				
5				
6				
7				
8				
9				
10				

CONFIDENTIAL

NO.	QUESTIONS AND INSTRUCTIONS	CODING CATEGORIES	SKIPS/FILTERS
**HOUSEHOLD CHARACTERISTICS**			
101	What is the <u>main</u> source of drinking water for members of your household?	**PIPED WATER**<br>PIPED INTO DWELLING=11<br>PIPED TO YARD/PLOT=12<br>PUBLIC TAP/STANDPIPE=13<br>TUBE WELL OR BOREHOLE=21<br>**DUG WELL**<br>PROTECTED WELL=31<br>UNPROTECTED WELL=32<br>**WATER FROM SPRING**<br>PROTECTED SPRING=41<br>UNPROTECTED SPRING=42<br>RAINWATER=51<br>TANKER TRUCK=61<br>CART WITH SMALL TANK OR JERRY CAN/CARTLESS VENDOR=71<br>SURFACE WATER (RIVER/DAM/LAKE/ POND/STREAM/CANAL)=81<br>BOTTLED WATER/DISPENSER WATER=91<br>SACHET (PURE) WATER=92<br>OTHER (SPECIFY) = 96<br>________________________<br>DON'T KNOW = 98<br>REFUSED TO SAY = 99	
102	What do you do to make your water safe for drinking?	BOILING=1<br>USE WATER FILTER (CERAMIC, CHARCOAL, SAND, COMPOSITE, ETC).=2<br>SEDIMENTATION (LET IT STAND AND SETTLE)=3<br>USE ALUM=4<br>DISINFECTION (WATERGUARD, BLEACH, CHLORINE)=5<br>USE BOTTLED WATER=6<br>USE SACHET (PURE WATER)=7<br>STRAIN THROUGH A CLOTH=8<br>DO NOT TREAT WATER=9<br>OTHER (SPECIFY) = 96<br>________________________<br>DON'T KNOW = 98<br>REFUSED TO SAY = 99	
103	What kind of toilet facility do members of your household usually use?	FLUSH OR POUR FLUSH TOILET=11<br>TRADITIONAL PIT LATRINE=21<br>VENTILATED IMPROVED PIT LATRINE (VIP)=22<br>NO FACILITY/BUSH/FIELD=61<br>OTHER (SPECIFY) = 96<br>________________________<br>DON'T KNOW = 98<br>REFUSED TO SAY = 99	IF NO FACILITY→105
104	Do you share this toilet facility with other households?	YES=1<br>NO=2<br>OTHER (SPECIFY) = 96<br>________________________<br>DON'T KNOW = 98<br>REFUSED TO SAY = 99	

CONFIDENTIAL

NO.	QUESTIONS AND INSTRUCTIONS	CODING CATEGORIES	SKIPS/FILTERS
PREFACE BEFORE QUESTIONS 105-111:<br>Does your household have:			
105	Electricity?	YES=1<br>NO=2<br>DON'T KNOW=98<br>REFUSED TO SAY=99	If NO→109
106	Connection to the National Grid?	YES=1<br>NO=2<br>DON'T KNOW=98<br>REFUSED TO SAY=99	
107	Solar power/inverter?	YES=1<br>NO=2<br>DON'T KNOW=98<br>REFUSED TO SAY=99	
108	Generator?	YES=1<br>NO=2<br>DON'T KNOW=98<br>REFUSED TO SAY=99	
109	A radio?	YES=1<br>NO=2<br>DON'T KNOW=98<br>REFUSED TO SAY=99	
110	A telephone/mobile telephone?	YES=1<br>NO=2<br>DON'T KNOW=98<br>REFUSED TO SAY=99	
111	A refrigerator?	YES=1<br>NO=2<br>DON'T KNOW=98<br>REFUSED TO SAY=99	
112	What type of fuel does your household <u>mainly</u> use for cooking?<br><br>PROBE FOR MAIN ONE<br><br>SELECT ONLY ONE	FIREWOOD/STRAW=1<br>PARAFIN/KAROSENE=2<br>NATURAL GAS=3<br>ELECTRICITY=4<br>CHARCOAL FROM WOOD=5<br>COAL, LIGNITE=6<br>DUNG=7<br>NO FOOD COOKED IN HOUSEHOLD=95<br>OTHER (SPECIFY)=96<br>____________<br>DON'T KNOW=98<br>REFUSED TO SAY=99	

CONFIDENTIAL

NO.	QUESTIONS AND INSTRUCTIONS	CODING CATEGORIES	SKIPS/FILTERS
113	MAIN MATERIAL OF FLOOR<br><br>RECORD OBSERVATION<br><br>SELECT MAIN MATERIAL	**NATURAL FOOR**<br>EARTH/SAND=1<br>**FINISHED FLOOR**<br>PARQUET OR POLISHED WOOD=2<br>VINYL OR ASPHALT STRIP=3<br>CERAMIC TILE=4<br>CEMENT=5<br>TERAZO=6<br>OTHER (SPECIFY)=96<br>___________________________	
114	MAIN MATERIAL OF THE ROOF<br><br>RECORD OBSERVATION.<br><br>SELECT MAIN MATERIAL	NO ROOF=1<br>**NATURAL ROOFING**<br>THATCH/PALM LEAF=2<br>MUD=3<br>**RUDIMENTARY ROOFING**<br>CORRUGATED IRON =4<br>ALUMINIUM SHEET=5<br>**FINISHED ROOFING**<br>ASBESTOS SHEET=6<br>CONCRETE=7<br>STEP TILES=8<br>WOOD=9<br>OTHER (SPECIFY)=96<br>___________________________	
115	MAIN MATERIAL OF THE EXTERIOR WALLS<br><br>RECORD OBSERVATION.<br><br>SELECT MAIN MATERIAL	**NATURAL WALLS**<br>CANE/PALM/TRUNKS=1<br>MUD=2<br>**RUDIMENTARY WALLS**<br>BAMBOO WITH MUD=3<br>STONE WITH MUD=4<br>PLYWOOD=5<br>CARDBOARD/CARTON=6<br>REUSED WOOD=7<br>**FINISHED WALLS**<br>CEMENT=8<br>STONE WITH LIME/CEMENT=9<br>BRICKS=10<br>CEMENT BLOCKS=11<br>WOOD PLANKS=12<br>OTHER (SPECIFY)=96<br>___________________________	
116	How many bedrooms do you have?	NUMBER OF ROOMS:__ __<br>DON'T KNOW=98<br>REFUSED TO SAY=99	

PREFACE BEFORE QUESTIONS 117-120:
DOES ANY MEMBER OF YOUR HOUSEHOLD OWN:

CONFIDENTIAL

NO.	QUESTIONS AND INSTRUCTIONS	CODING CATEGORIES	SKIPS/FILTERS
117	A bicycle?	YES=1<br>NO=2<br>DON'T KNOW=98<br>REFUSED TO SAY=99	
118	A motorcycle or motor scooter?	YES=1<br>NO=2<br>DON'T KNOW=98<br>REFUSED TO SAY=99	
119	A car or truck?	YES=1<br>NO=2<br>DON'T KNOW=98<br>REFUSED TO SAY=99	
120	A boat or canoe with a motor?	YES=1<br>NO=2<br>DON'T KNOW=98<br>REFUSED TO SAY=99	
PREFACE BEFORE QUESTIONS 12-126:<br>DOES ANY MEMBER OF YOUR HOUSEHOLD OWN:			
121	Cows?	YES=1<br>NO=2<br>DON'T KNOW=98<br>REFUSED TO SAY=99	
122	Goats/Sheep?	YES=1<br>NO=2<br>DON'T KNOW=98<br>REFUSED TO SAY=99	
123	Poultry (e.g., ducks, chickens)?	YES=1<br>NO=2<br>DON'T KNOW=98<br>REFUSED TO SAY=99	
124	Dogs?	YES=1<br>NO=2<br>DON'T KNOW=98<br>REFUSED TO SAY=99	
125	Other animals (camels, horses, donkeys)?	YES=1<br>NO=2<br>DON'T KNOW=98<br>REFUSED TO SAY=99	

CONFIDENTIAL

NO.	QUESTIONS AND INSTRUCTIONS	CODING CATEGORIES	SKIPS/FILTERS
126	Does your household have any mosquito nets that can be used while sleeping?	YES=1<br>NO=2<br>DON'T KNOW=98<br>REFUSED TO SAY=99	

NO.	QUESTIONS AND INSTRUCTIONS	CODING CATEGORIES	SKIPS/FILTERS
**Support for Orphans and Vulnerable Children**			
127	DO NOT READ: CHECK COLUMN G IN THE HOUSEHOLD SCHEDULE<br><br>ANY CHILD AGE 0-17 years?	NUMBER OF CHILDREN (0-17 YRS)<br>[ ]	IF '00' NONE → END THE INTERVIEW<br><br>IF AT LEAST ONE CHILD → 128
128	DO NOT READ: CHECK COLUMN 18 IN THE HOUSEHOLD SCHEDULE<br>Any sick adult age 18 -64 years?	YES=1<br>NO=2	If YES → 131
129	DO NOT READ: CHECK COLUMN 25 IN THE HOUSEHOLD SCHEDULE<br>Any child whose mother has died or is very sick?	YES=1<br>NO=2	If YES → 131
130	DO NOT READ: CHECK COLUMN 25 IN THE HOUSEHOLD SCHEDULE<br><br>Any child whose father has died or is very sick	YES=1<br>NO=2	If YES → 131
131	Record names, ages of children between 0 -17 years who are identified above as having a mother and/father who has died or has been very sick		

131	Record names, ages of children between 0 -17 years who are identified above as having a mother and/father who has died or has been very sick			
		CHILD (1)	CHILD (2)	CHILD (3)

CONFIDENTIAL

	NAME			
	LINE NUMBER (FROM COLUMN A)			
	AGE (FROM COLUMN G)			
INTERVIEWER SAY: "I would like to ask you about any formal, organized help or support for children that your household may have received for which you did not have to pay. By formal, organized support, I mean help provided by someone working for a program. This program could be government, private, religious, charity, or community-based."				
132	Now , I would like to ask you about the support your household received for (NAME) In the last 12 months, has your household received any medical support for (NAME), such as medical care, supplies, or medicine, for which you did not have to pay?	YES=1<br>NO=2<br>DON'T KNOW=98	YES=1<br>NO=2<br>DON'T KNOW=98	YES=1<br>NO=2<br>DON'T KNOW=98
133	In the last 12 months, has your household received any emotional or psychological support for (NAME), such as companionship, counselling from a trained counsellor, or spiritual support, which you received at home and for which you did not have to pay?	YES=1<br>NO=2<br>DON'T KNOW=98	YES=1<br>NO=2<br>DON'T KNOW=98	YES=1<br>NO=2<br>DON'T KNOW=98
134	Did your household receive any of this emotional or psychological support for (NAME) in the past 3 months?	YES=1<br>NO=2<br>DON'T KNOW=98	YES=1<br>NO=2<br>DON'T KNOW=98	YES=1<br>NO=2<br>DON'T KNOW=98
135	In the last 12 months, has your household received any material support for (NAME), such as clothing, food, or financial support, for which you did not have to pay?	YES=1<br>NO=2<br>DON'T KNOW=98	YES=1<br>NO=2<br>DON'T KNOW=98	YES=1<br>NO=2<br>DON'T KNOW=98
136	Did your household receive any of this material support for (NAME) in the past 3 months?	YES=1<br>NO=2<br>DON'T KNOW=98	YES=1<br>NO=2<br>DON'T KNOW=98	YES=1<br>NO=2<br>DON'T KNOW=98
137	In the last 12 months, has your household received any social support for (NAME), such as help in household work, training for a caregiver, or legal services, for which you did not have to pay?	YES=1<br>NO=2<br>DON'T KNOW=98	YES=1<br>NO=2<br>DON'T KNOW=98	YES=1<br>NO=2<br>DON'T KNOW=98
138	Did your household receive any of this social support for			

CONFIDENTIAL

	(NAME) in the past 3 months?			

If child is 0-4 years, Go to end and continue to next child if other children whose mother and/or father has died or is very sick.<br>If child is 5-17 years, go to				
139	Has (NAME) ever attended school?	YES=1<br>NO=2<br>DON'T KNOW=98	YES=1<br>NO=2<br>DON'T KNOW=98	YES=1<br>NO=2<br>DON'T KNOW=98
140	What is the highest level of school (NAME) completed? Probe to get specific education level.	Nursery………………..1<br>Primary ………………..2<br>Post primary/vocation …….3<br>Secondary…………..4<br>College ………….5<br>University…………………6<br>Don't know ………………98	Nursery………………..1<br>Primary ………………..2<br>Post primary/vocation …….3<br>Secondary…………..4<br>College ………….5<br>University…………………6<br>Don't know …………98	Nursery………………..1<br>Primary ………………..2<br>Post primary/vocation …….3<br>Secondary…………..4<br>College ………….5<br>University…………………6<br>Don't know …………98
141	In the last 12 months, has your household received any support for (NAME)'s schooling, such as allowance, free admission, books, or supplies, for which you did not have to pay?	YES=1<br>NO=2<br>DON'T KNOW=98	YES=1<br>NO=2<br>DON'T KNOW=98	YES=1<br>NO=2<br>DON'T KNOW=98
Interviewer says: "Thank you for the information regarding (NAME).<br>IF THERE IS ANOTHER CHILD 0-17 YEARS IN THE HOUSEHOLD WHO HAS BEEN IDENTIFIED UNDER QUESTION 129 AND QUESTION 130 AS HAVING A MOTHER/FATHER WHO HAS DIED OR IS VERY SICK BESIDES (NAME) → CONTINUE FROM QUESTION 132 AND ASK ABOUT THE NEXT CHILD.<br>Interviewer says: "Next , I would like to ask you about (NAME)"<br>IF NO OTHER CHILDREN, END THE HOUSEHOLD INTERVIEW.				
INTERVIEWER SAY: "This is the end of the household survey. Thank you very much for your time and for your responses."				

CONFIDENTIAL
